# Anti-inflammatory role of Gpnmb in adipose tissue of mice

**DOI:** 10.1038/s41598-021-99090-6

**Published:** 2021-10-04

**Authors:** Bernadette Nickl, Fatimunnisa Qadri, Michael Bader

**Affiliations:** 1 Max-Delbrück-Center for Molecular Medicine in the Helmholtz Association, Robert-Rössle-Str. 10, 13125 Berlin, Germany; 2 Berlin Institute of Health at Charité – Universitätsmedizin Berlin, 10178 Berlin, Germany; 3 German Center for Cardiovascular Research (DZHK), Partner Site Berlin, Berlin, Germany; 4 Charité University Medicine, 10117 Berlin, Germany; 5 Institute for Biology, University of Lübeck, 23538 Lübeck, Germany

**Keywords:** Inflammation, Metabolic disorders, Obesity

## Abstract

Obesity can cause a chronic, low-grade inflammation, which is a critical step in the development of type II diabetes and cardiovascular diseases. Inflammation is associated with the expression of glycoprotein nonmetastatic melanoma protein b (Gpnmb), which is mainly expressed by macrophages and dendritic cells. We generated a Gpnmb-knockout mouse line using Crispr-Cas9 to assess the role of Gpnmb in a diet-induced obesity. The absence of Gpnmb did not affect body weight gain and blood lipid parameters. While wildtype animals became obese but remained otherwise metabolically healthy, Gpnmb-knockout animals developed, in addition to obesity, symptoms of metabolic syndrome such as adipose tissue inflammation, insulin resistance and liver fibrosis. We observed a strong Gpnmb expression in adipose tissue macrophages in wildtype animals and a decreased expression of most macrophage-related genes independent of their inflammatory function. This was corroborated by in vitro data showing that Gpnmb was mostly expressed by reparative macrophages while only pro-inflammatory stimuli induced shedding of Gpnmb. The data suggest that Gpnmb is ameliorating adipose tissue inflammation independent of the polarization of macrophages. Taken together, the data suggest an immune-balancing function of Gpnmb that could delay the metabolic damage caused by the induction of obesity.

## Introduction

Obesity has become endemic in the Western world, mainly caused by poor diet and sedentary lifestyle, and is a major risk factor for the development of cardiovascular diseases. Gpnmb is a protein that is heavily induced in inflammatory diseases, while endogenous Gpnmb expression in healthy tissue is very low^1,2^. In humans, GPNMB serum levels are associated and contribute to obesity and metabolic parameters such as hip circumference, body mass index and insulin resistance^3^. In screenings for differentially expressed genes induced by high fat diet, Gpnmb was found among the highest expressed genes in white adipose tissue of mice and humans^4,5^. Gpnmb is upregulated in adipose tissue by genetically^6^ and diet-induced^7^ obesity.

Gpnmb is a type I transmembrane protein that is heavily N-glycosylated and bears a short cytoplasmic and a major extracellular domain^2,8^. Mouse, rat and human orthologues of Gpnmb, also called DC-HIL^9^, Osteoactivin^10^ and HGFIN^11^, respectively, are very similar in their structure. With its extracellular heparin and integrin binding domains, Gpnmb has the capacity to bind several cell types like vascular endothelial cells, keratinocytes, melanoma cells and fibroblasts^9,12^. Soluble, extracellular Gpnmb is produced by ectodomain shedding by ADAM10^13^. Soluble Gpnmb found in plasma is likely to exert different functions than the full-length, transmembrane version. CD44^3,14^ and syndecan-4 were identified as receptors for Gpnmb, binding of the latter can inhibit activated T cells^15,16^.

Gpnmb is expressed by mature macrophages derived from bone marrow^1,17^, by newly infiltrating macrophages into diseased tissue such as liver fibrosis or kidney injury^18–20^ and by resident, tissue-specific macrophages of liver, brain or the peritoneum^1,20–23^. Macrophages display a high degree of plasticity in adjusting their phenotype to their respective environment. Whereas Gpnmb induction was detected in both pro- and anti-inflammatory macrophage phenotypes^22,24–26^, the effect of Gpnmb expression seems to be anti-inflammatory^1,7,14,27^ and occurs after the acute phase of an inflammation^28^, suggesting a role in repair and remodeling processes.

Intriguingly, Gpnmb expression was repeatedly found in macrophages that are characterized by lipid overload. For instance, Gpnmb is expressed by lipid-loaded macrophages in obesity^5,7^, atherosclerosis^18^, Gaucher-, Niemann-Pick^29^, Parkinson´s^30^ as well as Alzheimer´s disease^31^. They all have in common, that lipid accumulation exceeds lysosomal capacity of macrophages that hence turn into foam cells. Even in renal tissue after acute kidney injury, Gpnmb localizes to cholesterol-containing vesicles^18^. Thus, Gpnmb function in this context could be related to macroautophagy^18^ or to lysosomal stress^32^.

The upregulation of Gpnmb in many diseases is often interpreted as disease-related and thus detrimental. However, the upregulation of Gpnmb might derive from infiltrating macrophages that exert anti-inflammatory and immune-balancing effects. We investigated the effects of Gpnmb in the inflammatory, macrophage-dependent disease obesity and anticipated an effect on body weight. Unexpectedly, we had to reject this hypothesis but detected a profound effect of Gpnmb deficiency on adipose tissue inflammation and insulin sensitivity in obese mice.

## Results

### Gpnmb reduces inflammation of adipose tissue

**Figure 1:**
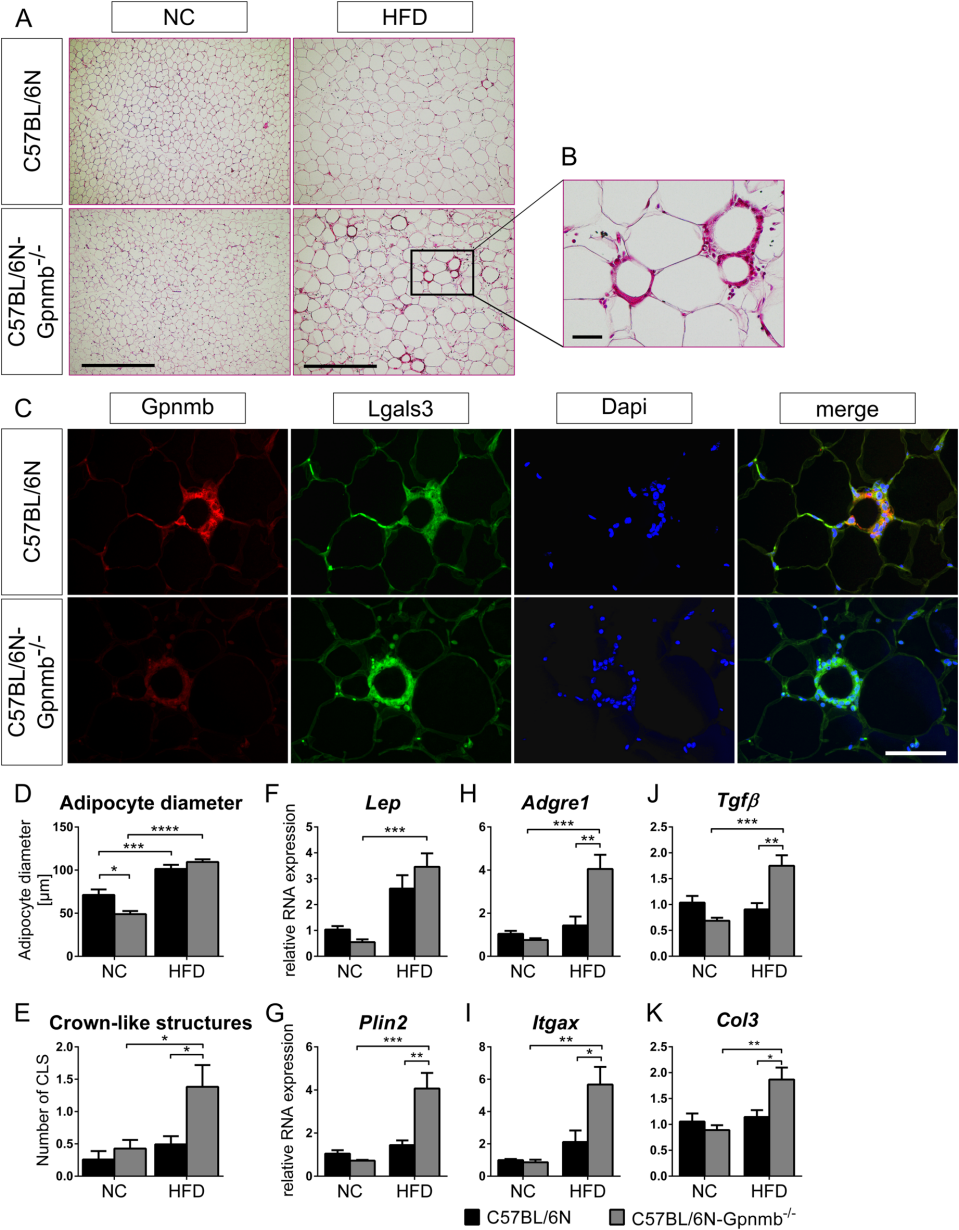
Analysis of epididymal adipose tissue of lean or HFD-fed, male Gpnmb^-/-^ and wildtype mice. (**A**) Representative pictures of hematoxylin and eosin staining of 10 µm sections of epididymal adipose tissue. Scale bar: 500 µm (**B**) Crown-like structure (CLS). Scale bar: 50 µm. (**C**) Gpnmb and macrophage localization in immunohistological stainings of 7 µm adipose tissue sections. Scale bar: 100 µm. (**D,E**) Analysis of histological adipose tissue sections. On average 11 microscopic views (1.56 mm^2^) were analyzed per animal. (**D**) The size of on average 37 cells was measured per microscopic view. (**F–K**) Transcript levels of inflammatory genes measured by qRT-PCR. Relative RNA expression was calculated using the 2^-∆∆Ct^ method, normalized to the housekeeping genes and to NC-fed, wildtype animals. NC: normal chow, HFD: high fat diet. (**A**–E): n = 6–7, (**F**–**K**) n = 5–7, mean ± SEM. Statistical differences were determined by a Two-way ANOVA with Bonferroni post-hoc tests; * *p* < 0.05; **p < 0.01; *** *p* < 0.001; **** *p* < 0.0001.

To assess the role of Gpnmb in the chronic, low-grade inflammatory disease obesity, we created a Gpnmb^-/-^ knockout mouse line on the C57Bl/6N inbred genetic background using Crispr-Cas9. As expected, Gpnmb could not be detected on protein level in healthy mouse organs but was detectable after enriching Gpnmb protein in primary bone marrow-derived macrophages. No signal was detected in cells derived from Gpnmb^-/-^ mice by Western Blot (Fig. S1). *Gpnmb* mRNA in organs from obese and normal chow (NC)-fed Gpnmb^-/-^ mice as well as soluble Gpnmb in plasma was hardly detectable (Fig. S2). Although *Gpnmb* transcription should remain unaffected by the knockout, we could still detect a drastic downregulation of its mRNA, probably due to efficient nonsense mediated decay^33,34^. This phenomenon was also shown previously in another Gpnmb-knockout mouse^35^. To establish a diet-induced obesity, male Gpnmb^-/-^ and wildtype mice were fed a high fat diet (HFD) for 16 weeks. Fat, liver and brain *Gpnmb* mRNA were increased by 16 weeks of HFD (Fig. S2). Plasma levels of circulating Gpnmb were not altered between lean female and male animals but increased in males after 16 weeks of HFD. Throughout the 16 weeks of feeding HFD, body weight and weight of adipose tissue increased similarly in both strains (Fig. S3A–F). The HFD-fed wildtype and Gpnmb^-/-^ mice started with a mean body weight of 23.92 ± 0.77 g and 24.84 ± 0.88 g, respectively, and reached a body weight of 38.27 ± 1.62 g and 40.45 ± 2.25 g. The NC-fed wildtype and Gpnmb^-/-^ mice weighed 32.74 ± 1.80 g and 30.34 ± 1.06 g on the day of the organ collection. HFD elevated blood levels of total as well as HDL cholesterol in both Gpnmb^-/-^ and wildtype mice, whereas triglyceride and LDL levels were not affected (Fig. S3G**–**J). Analysis of epididymal adipose tissue sections revealed an increased adipocyte diameter induced by HFD, resulting in the expected hypertrophy of adipocytes (Fig. 1A,B,D). In addition, obesity was confirmed by decreasing levels of *Adipoq, Cebpa* and *Slc2a4* mRNAs (Fig. S4A–C) that are known to be inversely correlated to adiposity^36,37^.

Adipose tissue inflammation ensues adipogenesis in the pathology of metabolic syndrome. One sign of adipose tissue inflammation are the so-called crown-like structures (CLS)^38^. Indeed, small isolated cells appeared widely dispersed between adipocytes in lean mice but formed aggregates surrounding adipocytes in obese mice. Those aggregates consisted of Lgals3-positive macrophages that were already turning into foam cells full of Plin2-positive lipid vesicles (Fig. S5A). Those CLSs were the source of Gpnmb expression in wildtype mice (Fig. 1C). CLSs occurred in higher frequency in obese Gpnmb^-/-^ animals (Fig. 1E), suggesting an inhibitory effect of Gpnmb on macrophage infiltration into adipose tissue. This was corroborated by mRNA data of macrophage and inflammation markers that were increased in adipose tissue of obese Gpnmb^-/-^ animals (Fig. S4). *Itgax* (also known as CD11c) and *Ccr2* are marker genes for recruited macrophages that would act pro-inflammatory; *Adgre1* (also known as F4/80), *Mrc1* (also known as CD206) and *Arg1* for resident adipose tissue macrophages that act anti-inflammatory^39^. *Abca1* and *Plin2* are marker genes for metabolically activated macrophages in response to obesity-associated cues. The expression of those genes except *Abca1* was increased in obese, Gpnmb^-/-^ animals. Moreover, the expression of several markers for fibrosis (Fig. 1F–K, Fig. S4P–R) as well as of *Cybb*, a marker for oxidative stress (Fig. S4D), were increased in obese Gpnmb^-/-^ animals. This upregulation of mRNAs for several genes which are specifically expressed in macrophages could be explained by the increased number of macrophages infiltrating adipose tissue in the absence of Gpnmb. Interestingly, the signal of the mannose receptor Mrc1 was located to small, isolated cells and only rarely to CLSs, corroborating its reported expression in resident macrophages (Fig. S5B–D)^39^. In both wildtype and Gpnmb^-/-^ mice, these single cells were unevenly distributed in adipose tissue, some areas rich with CLSs were completely devoid of Mrc1 signal (Fig. S5C). Obese Gpnmb^-/-^ animals showing increased levels of *Mrc1* mRNA suggest that Gpnmb deficiency might even increase the number of those resident cells. Surprisingly, we could hardly detect CLSs in wildtype mice, suggesting that those animals have not yet reached this step of adipose tissue inflammation.

### Gpnmb might ease insulin resistance

**Figure 2:**
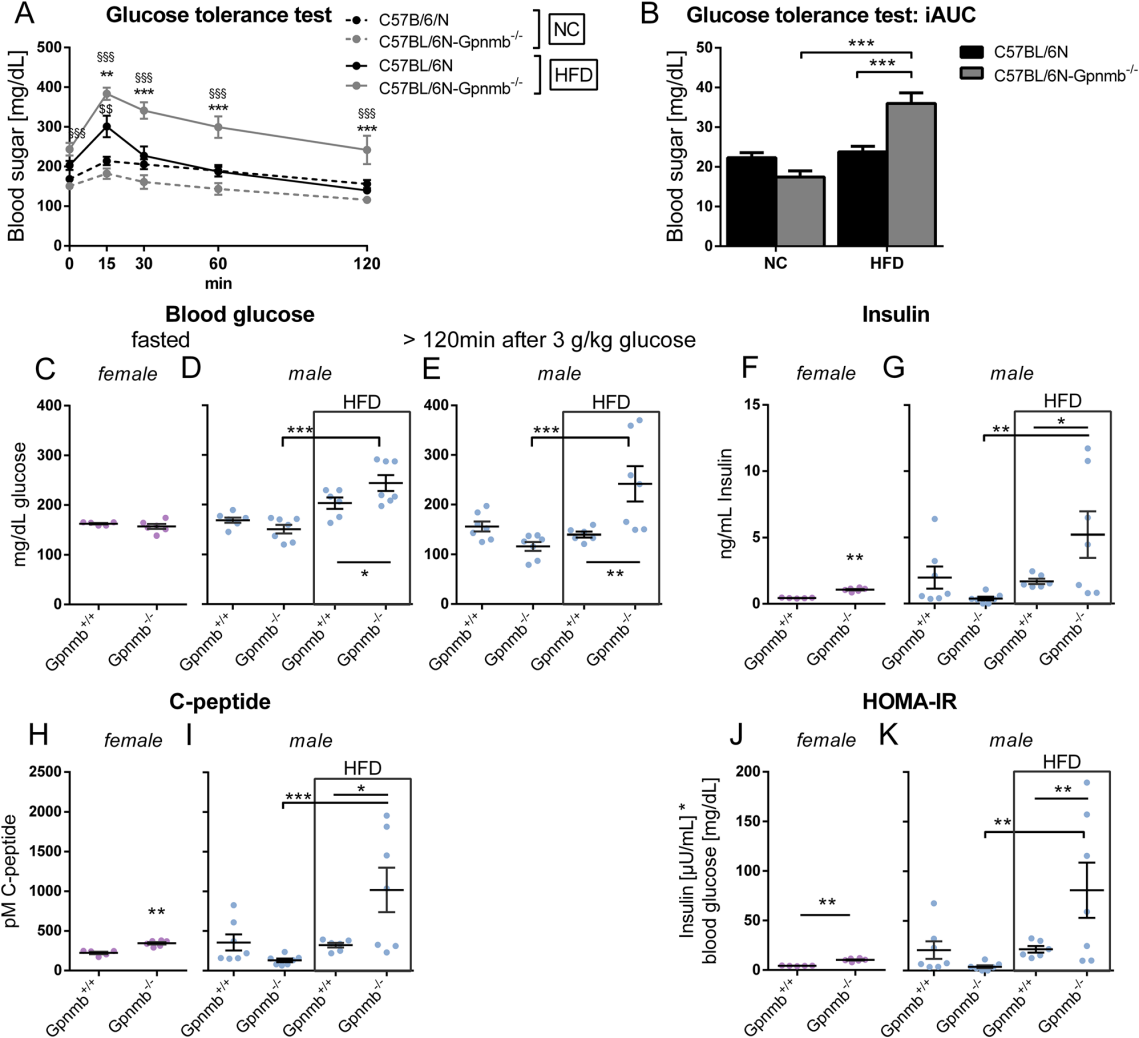
Insulin resistance of lean or HFD-fed Gpnmb^-/-^ and wildtype mice. (**A**) Blood glucose time line of oral glucose tolerance test conducted in 6 h fasted animals and (**B**) its respective integrated area under the curve (iAUC). (**C–E**) Blood glucose of (**A**) in comparison to female control animals. (**F,I**) ELISA of insulin and C-peptide in plasma. (**J–K**) Homeostatic model assessment for insulin resistance (HOMA-IR), a product of insulin and fasted blood sugar. NC: normal chow, HFD: high fat diet. n = 6–7, mean ± SEM. Statistical differences were determined by a non-parametric t-test/Mann Whitney test for female animals (**C**,**F**,**H**,**J**) and by a Two-way ANOVA with Bonferroni post-hoc tests for male animals (**A**,**B**,**D**,**E**,**G**,**I**,**K**). (**A**) * C57BL/6N (16 weeks HFD) vs. C57BL/6N-Gpnmb-/- (16 weeks HFD); $ C57BL/6N (16 weeks HFD) vs. C57BL/6N (NC); § C57BL/6N-Gpnmb-/- (16 weeks HFD) vs. C57BL/6N-Gpnmb-/- (NC). For all graphs: * *p* < 0.05; **/$$ *p* < 0.01; ***/§§§ *p* < 0.001.

Adipose tissue inflammation is considered a causative factor for insulin resistance and hence an impaired clearance of glucose in the blood^38,40,41^. Glucose levels peaked distinctly within 15 min after an oral glucose dose of 3 g/kg body weight in HFD-fed but not in lean animals (Fig. 2A–E), showing that insulin sensitivity was deteriorated by HFD. In obese Gpnmb^-/-^ animals, the peak was further increased and glucose clearance delayed compared to wildtype controls. Some of the obese Gpnmb^-/-^ mice still exhibited hyperglycemia (> 300 mg/dL^42^) 120 min after the oral dose of glucose (Fig. 2E). In line with glucose levels, insulin and C-peptide levels were increased in HFD-fed, Gpnmb^-/-^ animals (Fig. 2F–I). However, all male animals had received an oral dose of glucose more than 2 h before blood extraction for ELISA measurements, which may have influenced insulin and C-peptide values. Therefore, another control group was added. Insulin levels were significantly increased in 6 h fasted, female Gpnmb^-/-^ animals, although these animals were still able to maintain similar baseline glucose levels as wildtype controls (Fig. 2C,F,H). Thus, already lean, female Gpnmb^-/-^ animals might be at the beginning of insulin resistance that could have been further enhanced by HFD, as indicated by the homeostatic model assessment for insulin resistance (HOMA-IR) values for male mice (Fig. 2J–K).

### Gpnmb prevents liver fibrosis

Diet-induced obesity induces a chronic low-grade inflammation of several tissues. Other organs such as liver, muscle and brain did not show the same level of inflammation induced by HFD in Gpnmb^-/-^ animals compared to white adipose tissue (Fig. S6). Still, obesity led in liver to a significant increase of macrophage marker *Adgre1* expression. Liver inflammation can be a result of steatosis, an ectopic fat deposition in liver. Indeed, histology showed large vacuoles full of fat in liver sections that appeared in both genotypes after 16 weeks of HFD (Fig. S7A). Fibrotic depositions stained by Sirius Red seemed more pronounced in obese Gpnmb^*-/-*^ animals, so we tested for liver damage markers in plasma. An increase of ALT in plasma was seen exclusively in obese Gpnmb^-/-^ animals (Fig. S7B) and indicated an ameliorating influence of Gpnmb on the development of liver damage. AST remained equal in all conditions, excluding general organ damage. In line with this, the expression of fibrotic genes like collagens and *Tgfβ* were upregulated by HFD in the liver of Gpnmb^-/-^ but not of wildtype animals (Fig. S7C). A key regulator of metabolism is AKT1/2/3/Protein Kinase B. Full activation of AKT is achieved by phosphorylation of serine 473 by mTORC2 (rictor mTOR complex), which is especially important in diabetes^43,44^. AKT phosphorylation in liver responded to HFD, but only in Gpnmb^-/-^ animals, and showed the same pattern as insulin resistance and fibrotic gene expression with lean Gpnmb^-/-^ mice having a reduced AKT-S473 phosphorylation that was significantly increased by HFD (Fig. S7D,E). Thus, HFD caused liver damage only in the absence of Gpnmb and not in wildtype animals.

### Gpnmb is secreted mainly by inflammatory macrophages

**Figure 3:**
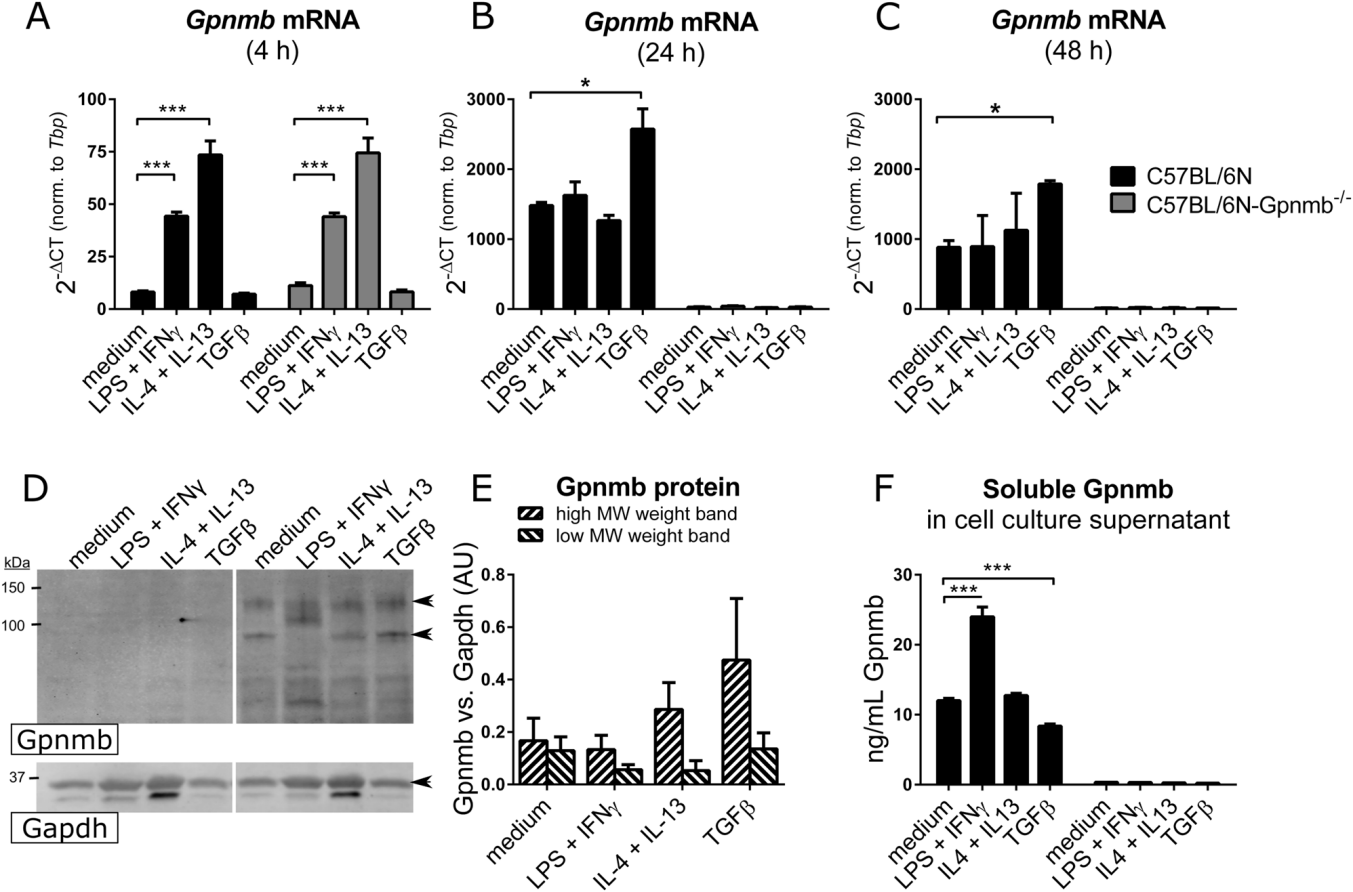
Gpnmb mRNA and protein levels of differently polarized Gpnmb^-/-^ and wildtype macrophages. Cells were treated with IL-4 and 20 ng/mL IL-13, or TGFβ, or LPS and IFNγ. (**A–C**) Transcript levels were measured by qRT-PCR after 4 h (**A**), 24 h (**B**) or 48 h (**C**). (**D–F**) Gpnmb protein expression and release of differently polarized macrophages after 48 h. (**D**) Exemplary Western blot of stimulated cell extracts. Undiluted lysates were loaded onto a SDS gel. Cropped image, the respective full-length blots are presented in Fig. S9. (**E**) Quantification of the two characteristic Gpnmb bands (indicated by arrows in **D**) of wildtype macrophage extracts, normalized to Gapdh signal as loading control. (**F**) Quantification of released soluble Gpnmb in cell culture supernatant by ELISA. n = 3, meaning BMDMs from three individual mice per group, mean ± SEM. Statistical differences were determined by a Two-way ANOVA with Bonferroni post-hoc tests; * *p* < 0.05; *** *p* < 0.001.

We suspected that Gpnmb is only secondarily affecting insulin and glucose metabolism in obesity via adipose tissue inflammation. Thus, its main effect would be to reduce the activation of macrophages in an inflammatory environment. To check this in vitro, mature macrophages derived from wildtype and Gpnmb^-/-^ bone marrow were polarized with a single dose of the cytokines IL-4 and IL-13 (anti-inflammatory M2a phenotype), TGFβ (reparative M2c phenotype), or IFNγ and LPS (pro-inflammatory M1 macrophages) for 4, 24 or 48 h, respectively. On mRNA level, *Gpnmb* was upregulated after 4 h in M1 and even higher in M2a macrophages from both wildtype and Gpnmb^-/-^ mice (Fig. 3A–C). This changed at 24 and 48 h, when *Gpnmb* mRNA increased ~ 100-fold only in bone marrow-derived macrophages (BMDMs) of wildtype mice. At these time points, *Gpnmb* was induced especially in TGFβ-polarized M2c macrophages, which was confirmed on protein level by Western blot (Fig. 3D,E). The extracellular fragment of Gpnmb in the supernatant was measured at 48 h; here a different picture was observed: most of Gpnmb protein was shed by inflammatory M1 macrophages whereas reparative M2c macrophages showed reduced Gpnmb shedding (Fig. 3F). Thus, the macrophages with the highest Gpnmb expression retained the protein indicating that Gpnmb expression and shedding underlie different regulatory mechanisms. Importantly, this means that Gpnmb might act rather para- or endocrine in inflammatory conditions versus a direct effect on the expressing cell in reparative conditions.

TNFα and IL-6 were heavily released after pro-inflammatory M1 stimulation; however the effect of Gpnmb expression on the release of those cytokines remained non-significant. In unstimulated or TGFβ-stimulated macrophages, TNFα and IL-6 could not be detected. We observed a mixed picture when assaying for mRNA expression of M1/M2a/M2c markers (Fig. S8). The main induction of inflammatory marker genes occurred after 4 h and declined afterwards. Pro-inflammatory genes were expressed only after M1 stimulation and were either increased (*Tnfα, Il1β*) or decreased (*Nos2, CD86*) in Gpnmb^-/-^ macrophages. The anti-inflammatory marker *Il10* was lowered by the absence of Gpnmb. Fatty acid/ metabolic genes were neither affected by inflammation status of the cell nor by the absence of Gpnmb (Fig. S8I–K). The expression of the anti-inflammatory marker gene *Arg1* was increased in the absence of Gpnmb, contradicting the hypothesis of an anti-inflammatory function of Gpnmb but corroborating the in vivo data from adipose tissue. CD86, a protein expressed by antigen-presenting cells to modulate T cell activity, shows an expression pattern opposite to the one observed in obesity in vivo, which might be accounted for by the lack of T cells in the culture. Importantly, the expression of *Gpnmb* peaked one day after the peak expression of inflammatory marker genes, suggesting a function distinct from the polarity of the cells.

## Discussion

Here we investigated the effects of Gpnmb expression in a model disease for macrophage-mediated inflammation. We successfully induced obesity in wildtype and Gpnmb^-/-^ animals but failed to induce the concomitant adipose tissue inflammation, pre-diabetes and liver damage that could be summarized as metabolic syndrome in wildtype animals. Gpnmb^-/-^ animals however displayed all those symptoms. Gpnmb was especially upregulated in adipose tissue macrophages, so called “crown-like structures”, which was described before^7^. Thus, Gpnmb was likely to play an important role in the development of low-grade inflammation in adipose tissue, which enhances the pathogenicity of obesity^4,5,7^. However, the exact cellular mechanism of Gpnmb buffering adipose tissue inflammation remains elusive. One possibility is a mild general anti-inflammatory effect of Gpnmb on macrophages, an effect that was reported several times^1,7,14,27^. However, the analysis of several inflammatory and metabolic marker genes that are related to macrophage properties in vivo and in vitro showed a mixed picture that could mean that Gpnmb acts either unspecifically on all macrophages or creates a polarity of macrophages that is distinct from the known M1/M2 paradigm, as was reported for microglia^45^.

Unexpectedly, wildtype animals did not suffer from obesity-related metabolic damage. One could speculate that Gpnmb contributes to the maintenance of a “healthy obese” phenotype even during obesity^46,47^. However, the down-regulation of *Adipoq* and *Slc2a4*, key regulators of adipose tissue inflammation and insulin sensitivity, suggests that wildtype mice were just delayed in their progression of metabolic syndrome^48–50^. Moreover, the worsened glucose clearance in wildtype animals already indicated the development of insulin resistance. As *Gpnmb* mRNA expression was higher in adipose tissue compared to other metabolically relevant tissues, we postulate that the deteriorated health of Gpnmb^-/-^ mice is a consequence of the lack of Gpnmb in adipose tissue, subsequently affecting the number of macrophages.

We could not detect a clear effect of Gpnmb on the polarization of macrophages, neither in vitro nor in adipose tissue. In vitro, we detected the highest Gpnmb expression in macrophages polarized by TGFβ, an important cytokine for suppression of immune response and tissue remodeling. This was also reported by others^51^, however we realized that this cell type contributes little to soluble Gpnmb that could be distributed in blood to other organs. Our in vitro data of increased shedding of Gpnmb in pro-inflammatory conditions could be translated into the inflamed adipose tissue of obesity, where adipose tissue inflammation is delayed in wildtype animals. Apart from M1/M2 marker genes, also the expression of other macrophage-related genes appeared to randomly increase in Gpnmb^-/-^ animals. Thus, the main action of Gpnmb could be to counteract the recruitment and survival of macrophages, acting thereby anti-inflammatory. Interestingly, it was reported that the metabolic alterations in adipose tissue might be caused less by an often reported M1/M2 switch of adipose tissue macrophages, but by the pure number of recruited macrophages upon obesity^52^. A one-fold increase in recruited immune cells was associated with a tenfold increase in TNFα, meaning that every cell exponentially influences the inflammatory environment. Phenotyping the macrophage population of adipose tissue revealed that Gpnmb expression is 200-fold induced in obesity-associated adipose tissue macrophages compared to lean mice and, strikingly, equally in M1 and M2 macrophages^53^. This suggests that the observed impact of Gpnmb in this study, increasing the number of macrophages, can be independent of M1/M2 polarization and sufficient to promote adipose tissue inflammation. Future studies should therefore examine the impact of soluble Gpnmb on migration of macrophages. Some studies already suggested that Gpnmb might be involved in this process. Gpnmb is a heparin-mediated integrin ligand for endothelial cells and could therefore be responsible for transendothelial migration of dendritic cells^9^, mesenchymal stem cells^24^ and other bone marrow progenitors as well as tumor cells, while sabotaging the migration of T cells^54^. Not only the heparin-binding PKD domain but also the integrin-binding RGD-motif, phosphorylation at its intracellular tail as well as the KLD domain are important for migration, invasion and metastasis of cancer cells^55–57^. On the other hand, it was also reported that Gpnmb is able to inhibit migration of cancer cells^58,59^, which would match the immune-dampening effect we observed.

Katayama et al*.* utilized a transgenic mouse model with increased expression of Gpnmb in adipocyte fatty acid–binding protein (aP2)-expressing cells (adipocytes and macrophages) and detected an improved hepatic fat deposition and fibrosis, confirming our data; but unaltered fat accumulation, inflammation in adipose tissue and insulin sensitivity, suggesting an endocrine role of Gpnmb from adipose tissue to liver^19^. The lack of impact on adipose tissue might be due to the diet: The amount of sugar and fat were similar in both diets, but the fat of Katayama et al*.*`s diet derived mostly from coconut oil, the HFD of this study was mostly composed of lard. Lard but not coconut oil effectively induces insulin resistance^60^ because of the substantial amounts of palmitic acid in lard^61^. Palmitic acid is the starting substance of the inflammatory compounds ceramide, sphingosine and S1P, that are connected to insulin resistance^62–65^. Moreover, palmitic acid can induce Gpnmb expression^7^, possibly accounting for the stronger effect on adipose tissue in this study. Gpnmb induction is apparently quite specific to some lipids, which was studied in the context of lipid-storage diseases. Shortly, Gpnmb induction can be associated to the accumulation of lipids such as palmitate, cholesterol, and glucosylceramide, possibly also by their alternative degradation products, such as the glucosylceramide metabolites glucosylsphingosine, sphingosine or S1P^7,66–68^. Thus, Gpnmb might react either directly to those lipids or indirectly via the lysosomal stress that is caused by them^18,29,32^. Importantly, the fat sources of both studies (lard and coconut oil) are able to induce liver steatosis that was indeed detected in this and Katayama et al*.*’s study^60^. An endocrine effect of Gpnmb was also proposed by Gong et al*.,* but in the other direction. In this case, hepatically-secreted Gpnmb increased fat accumulation in adipose tissue and insulin resistance^3^, an effect that is opposite to our findings. However, Gong et al*.* concentrated on soluble Gpnmb that was not produced in adipose tissue. As mentioned before, soluble and full-length Gpnmb might have different functions, as we observed Gpnmb shedding upon pro-inflammatory stimuli and retention upon anti-inflammatory stimuli. Moreover, Gpnmb function was blocked using an antibody^3^, in contrast to us using a complete Gpnmb knockout. Importantly, it was shown before that blocking Gpnmb using an antibody can modify Gpnmb phosphorylation and induce maturation and a higher inflammatory potential of the Gpnmb-expressing cell^23^.

A connection between Gpnmb and insulin levels was described before: In obese OLETF rats, *Gpnmb* transcript levels increases in adipose tissue of obese compared to lean rats^19^. Also, patients with type II diabetes exhibit elevated GPNMB plasma levels compared to patients with normal glucose tolerance^19^ and GPNMB serum levels are associated to insulin resistance^3^. Our study indicates that those high Gpnmb levels can be a beneficial, compensatory response as the absence of Gpnmb aggravated plasma glucose and insulin levels. Insulin resistance or pre-diabetes are a permanent inflammatory condition that can increase the risk for multiple other diseases. Thus, Gpnmb levels can be relevant in patients of metabolic syndrome, and the proposed advantage of Gpnmb expression as well as potential clinical implications are worth further studying.

## Methods

### Animals

All mouse lines were kept in specific pathogen-free conditions in the animal care facility of the Max Delbrück Center for Molecular Medicine in the Helmholtz Association (MDC), Berlin, according to the German Animal Protection Law. All animal experiments were approved by the ethical committee of the local government (LaGeSo, Berlin), license number G0018/16. All experiments were performed in accordance with relevant guidelines and regulations. This study is reported in accordance with the ARRIVE guidelines. Mice were housed at a light/dark cycle of 12 h each.

Gpnmb^-/-^ mice were generated in cooperation with the Transgenic animal core facility at the MDC. The first base after the start codon ATG of the gene *Gpnmb* was deleted with Crispr-Cas9 technology in the C57BL/6N background strain (SI Methods). Gpnmb^-/-^ mice were held homozygously knockout for *Gpnmb* alleles and were compared to the C57BL/6N wildtype strain (Charles River, Sulzfeld, Germany) in animal experiments.

### Induction of obesity

To induce obesity, 10–12 weeks old, male Gpnmb^-/-^ mice and their wildtype controls were fed a high fat diet (HFD), with 60% calories from fat for 16 weeks (SI Methods). To minimize stress and the risk of rivalry fights, only 3–4 mice were held per cage. Body weight, consumed chow and water was measured twice a week. Once a week, the health status of each mouse was evaluated with the aid of a score sheet.

### Body composition analysis

A non-invasive body composition analysis was conducted to determine the percentage of water, fat and dry substance of the mice. Therefore, mice were fasted for 4 h, starting at 8am. The measurement was performed with a nuclear magnetic resonance (NMR) spectrometer (LF90II, Bruker, Billerica, USA) in the Pathophysiology core facility of the MDC, Berlin. Each measurement lasted about 3 min in a warm-temperate chamber, rendering anesthesia unnecessary.

### Glucose tolerance test

An oral glucose tolerance test was conducted in 6 h fasted mice (food removed at 8am) to detect changes in glucose metabolism. The tail was locally anesthetized with a 0.25% bupivacaine solution to prevent pain. Then, a blood sample was collected from the tail vein with the ACCU-Chek glucometer (Mannheim, Germany) to determine glucose levels. Mice were given one dose of glucose (3 g/kg at a concentration of 0.5 mg/µL) with an oral gavage and blood glucose was measured after 15, 30, 60, and 120 min.

### Measurement of lipids and liver parameters in plasma

Plasma samples from mice were diluted in ddH_2_O and triglycerides, cholesterol, HDL, LDL, alkaline phosphatase (ALP), aspartate aminotransferase (AST) and alanine aminotransferase (ALT) levels were analyzed in the Pathophysiology core facility of the MDC, Berlin, with an AU480 Chemistry Analyzer (Beckman Coulter, Brea, USA).

### Immunohistochemistry and tissue staining

Histology was performed as described before^35^. Primary antibodies used were Plin2: Fitzgerald # 20R-AP002 diluted 1:500; CD31: Abcam #ab28364 diluted 1:160; Gpnmb: R&D #AF2550 diluted 1:100; Lgals3: Enzo lifescience #CLO49P, diluted 1:200 in PBS. Details about the procedure can be found in the SI Methods.

### Cell culture

Bone marrow-derived macrophages (BMDMs) were obtained from tibias and femurs of 10- to 20-week-old mice. The epiphyses were cut off and the diaphysis was flushed with DMEM. The bone marrow was filtered through a 40 µm cell strainer to remove bone particles. Cells were incubated in Red Blood Cell Lysis buffer for 1 min at room temperature to remove erythrocytes. The reaction was stopped with addition of 10 mL of DMEM. Cells were cultured in complete RPMI1640 medium (RPMI1640 containing GlutaMAX, 100 U/ml penicillin/streptomycin, 10% fetal bovine serum and 30 ng/mL recombinant macrophage colony stimulating factor (M-CSF, Peprotech) or 33% L929 conditioned medium). Cells were seeded at a number of 1.5–2 × 10^6^ per well of a 6-well plate and cultured at 37 °C and 5% CO_2_. After three days, half of the medium was replaced by fresh complete medium. Differentiated macrophages were used at day 7. BMDMs were subsequently treated for 4, 24 or 48 additional hours with polarizing conditions as follows: 50 ng/mL LPS, 20 ng/mL IFN-γ; 20 ng/mL IL-4; 20 ng/mL IL-13 or 20 ng/mL TGFβ (all recombinant cytokines were obtained from Peprotech).

### qRT-PCR

RNA was isolated from tissues and transcript levels were measured as described in the SI Methods. Primer sequences are listed in Table S1.

### Western blotting

Standard Western blotting was performed using the antibodies Gpnmb: R&D #AF2330, Gapdh: Cell Signaling #2118; AKT (pan): Cell Signaling #4691; pAKT (Ser473): Cell Signaling #4060. Details of the procedures can be found in SI Methods.

### Statistical analysis

All data were subjected to statistical analysis using the GraphPad Prism 5 or 6 software (GraphPad Software Inc.). Data are expressed as mean ± SEM. A non-parametric t-test/Mann Whitney test was applied for comparisons between independent pairs of means. Two variables of interest were analyzed by a Two-way One-way analysis of variance (ANOVA) with Bonferroni post-test. Differences between two groups with a *p*-value of < 0.05 were considered to be statistically significant.

## Supplementary Information

Supplementary Information.

## Abbreviations

ALT: Alanine aminotransferase
AST: Aspartate aminotransferase
BMDMs: Bone marrow-derived macrophages
CLS: Crown-like structure
Gpnmb: Glycoprotein nonmetastatic melanoma protein B
HFD: High fat diet
IFNγ: Interferon γ
LPS: Lipopolysaccharide
MDC: Max Delbrück Center for Molecular Medicine in the Helmholtz Association
NC: Normal chow
TGFβ: Transforming growth factor β
