# Supplementary material for: Anti-inflammatory role of Gpnmb in adipose tissue of mice
Source: Sci Rep. 2021 Oct 4;11:19614. doi: 10.1038/s41598-021-99090-6 (PMC8490452; doi:10.1038/s41598-021-99090-6)
Supplement: Supplementary file 1 — Supplementary Information. [file 41598_2021_99090_MOESM1_ESM.pdf]

scientific reports

Supplementary Information for

Anti-inflammatory role of Gpnmb in adipose tissue of mice

Bernadette Nickl[A,B], Fatimunnisa Qadri[A], Michael Bader[A, B, C, D, E*]

Affiliations:

[A]Max-Delbrück-Center for Molecular Medicine in the Helmholtz Association, Robert-Rössle-Str. 10, 13125 Berlin, Germany
[B]Berlin Institute of Health at Charité – Universitätsmedizin Berlin, 10178 Berlin, Germany
[C]German Center for Cardiovascular Research (DZHK), Partner Site Berlin, Germany
[D]Charité University Medicine, 10117 Berlin, Germany
[E]University of Lübeck, Institute for Biology, 23538 Lübeck, Germany
*Corresponding author: Michael Bader
Email: mbader@mdc-berlin.de

This PDF file includes:

- **Supplemental Methods**
  - SI Animals: Generation of a *Gpnmb*-knockout mouse
  - SI Animals: Food Composition
  - SI Western Blotting
  - SI qRT-PCR; Table S1: Primer sequences
  - SI Immunohistochemistry and Tissue Staining
- **Supplemental Figures**
  - Figures S1 to S8
  - Figures S9 to S11 (Full-length Western Blots)
- **References**

Supplemental Methods

SI Animals: Generation of a Gpnmb-knockout mouse

The single guide RNA (sgRNA, 5´-GCAGGCGCTCGGAGTCAGCA) targeting *Gpnmb* was inserted as double-stranded DNA-oligonucleotide into the pX330 vector (pX330-U6-Chimeric_BB-CBh-hSpCas9, Addgene plasmid #42230) [1] that contains guide RNA and Cas9 expression cassettes. Positively transformed bacterial clones were selected by digestion with BbsI. Bacteria of one positive clone were expanded and the respective DNA was isolated with a PureYield™ Plasmid Maxiprep System and test sequenced. RNA was synthesized from the pX330 vector by *in vitro* transcription with a T7 polymerase. The resulting *Crispr* RNA targeting *Gpnmb* was microinjected together with *Cas9* mRNA and the single stranded DNA (ssDNA) template into the pronucleus of C57BL/6N zygotes. Those were subsequently implanted into foster mothers and the offspring was genotyped. The donor ssDNA (5'-C*T*G*T*GACTCCTGGTGGATGGGACTGGGGAGTCAGAGTCAAGCCCTGACTGGTTGCAGGCGCTCGGAGTCAGCATGAAAGTCTCTGCGGGGTCCTG*G*G*A*T, *= phosphorothioate rest at the respective base) was implemented as template to aim for homology-directed repair.

SI Animals: Food Composition

The calories from normal chow (V1124-300, Ssniff, Germany) derived to 53% from carbohydrates, 36% from protein and 11% from fat. This diet consisted of 34.9% starch, 22% protein and 4.5% fat (w/w).

The calories from high fat diet (MD.06414, Envigo, USA) derived to 18.3% from carbohydrates, 24.4% from protein and 60.3% from fat. This diet consisted of 27.3% carbohydrates, 23.5 from protein and 34.3% from fat (w/w).

SI Western Blotting

To extract proteins from cells, culture plates were washed twice with ice-cold PBS and scraped with an adequate volume of RIPA lysis buffer (Cell Signaling) containing Complete protease inhibitors and PhosStop phosphatase inhibitors (Roche). Cells or organ tissues were collected in FastPrep tubes containing 5 beads (skin: 10 beads) and homogenized with an adequate volume of RIPA buffer, using the FastPrep-24 instrument. Sample homogenates were sonicated for 30 sec and incubated at 4 °C for 30 min with intermittent mixing. Cell debris was removed by centrifugation at 13,000*g for 10 min at 4 °C. The concentration of proteins was determined using a bicinchoninic acid kit. A standard series of bovine serum albumin was used as reference.

SDS-PAGE was performed using the Bio-Rad electrophoresis system. Separating gels containing 10% acrylamide were casted and overlaid with the stacking gel containing 5% acrylamide. Protein samples were adjusted to an equal amount of 10-50 µg protein with ddH_2O and one part of 4x Roti-load reducing loading buffer in a total volume of 20 µL. Recombinant mouse Gpnmb (antibodies-online GmbH) was used as a positive control. Samples were denatured at 95 °C for 5 min and loaded onto the gel. Electrophoretically separated proteins were transferred from the SDS-PAGE gel onto a polyvinylidene difluoride (PVDF) membrane using a wet transfer system from Bio-Rad. The blotting was performed at 0.28 mA for 2.5 h at 4 °C. The blotted membrane was blocked with Odyssey blocking buffer for a minimum of 30 min at room temperature. The primary antibodies were diluted 1:1000 in a mix of 2.5 mL Tris-buffered saline containing 0.5% Tween-20 (TBST) and 2.5 mL of Odyssey blocking buffer, and incubated at 4 °C overnight. The membrane was incubated with Odyssey IRDye anti-mouse/rabbit/goat secondary antibody, diluted 1:10,000 in TBST, for 2 h at room temperature. After 3-4 washes 10 min each with 20 mL TBST, the membrane was scanned using an Odyssey infrared imaging system. The signals were analyzed using Image Studio Lite Software Version 5.2.5 (2015, LI-COR Bioscience, Lincoln, USA). The same membrane was incubated with anti-glyceraldehyde-3-phosphate dehydrogenase (Gapdh) antibody to normalize the signal on the membrane.

SI qRT-PCR:

Organs were collected in FastPrep tubes containing five beads and homogenized with 1 mL of TriFast peqGOLD (Peqlab) or Trizol (Invitrogen) reagent using a FastPrep™-24 instrument. Cells were lysed in the cell culture dish with 1 mL TRIzol or TriFast, scraped, transferred to a FastPrep tube containing five beads and homogenized using a FastPrep™-24 instrument. Thereafter, the manufacturers` instructions of Trizol or TriFast were followed.

To remove residual DNA, 1.5-4 µg of RNA were treated with DNase digestion mix at 37°C for 20 min. After heat-inactivating the enzyme at 75°C, the mix was quickly chilled on ice to avoid re-formation of RNA secondary structures. Secondly, 1µg of RNA was reverse transcribed into complementary DNA with reverse transcriptase (Invitrogen, Promega). qPCR was performed using SYBR green.
The Ct values were normalized to the housekeeping gene(s) Tbp and Gapdh. If two housekeeping genes were used, the geometric mean of the Ct values was used. Data were analyzed according to the $2^{-\Delta\Delta Ct}$ method to show relative RNA expression or with $2^{-\Delta Ct}$ to show RNA expression relative to the housekeeper.

Table S1: Primer sequences
All oligonucleotides were synthetized by Biotez Berlin Buch GmbH, Berlin and delivered in a lyophilized state. The primers were diluted in water to a working concentration of 5 pmol/µl. The primers are specific for the respective mouse gene.

Abbreviation	Protein	Sequence 5’→ 3'	Reference
Abca1	ATP-binding cassette transporter	CCCAGAGCAAAAAGCGACTC<br>GGTCATCATCACTTTGGTCCTTG	2
Adgre1	Adhesion G Protein-Coupled Receptor E1 (also known as F4/80, Emre1)	CATCCAGCCAAAGCAGAAGT<br>CAGCTGCAGACTGTGTGTGT	3
Adipoq	Adiponectin	TGTTCCTCTTAATCCTGCCCA<br>CCAACCTGCACAAGTTCCCTT	4
Arg1	Arginase1	CTCCAAGCCAAAGTCCTTAGAG<br>AGGAGCTGTCATTAGGGACATC	3
Ccl5	chemokine (C-C motif) ligand 5	GCTGCTTTGCCTACCTCTCC<br>TCGAGTGACAAACACGACTGC	
Ccr2	chemokine (C-C motif) receptor 2	ATCCACGGCATACTATCAACATC<br>TCGTAGTCATACGGTGTGGTG	5
Cd36	Cluster of differentiation 36	TTTCCTCTGACATTTGCAGGTCTA<br>AAAGGCATTGGCTGGAAGAA	2
Cd86	Cluster of differentiation 86 (also known as B7-2)	TCTCCACGGAAACAGCATCT<br>CTTACGGAAGCACCCATGAT	6
Cebpα	CCAAT Enhancer Binding Protein α	GTGGACAAGAACAGCAACGAGTA<br>CATTGTCACTGGTCAACTCCAG	
Chil3	Chitinase-like protein 3 (also known as Ym1)	CCCCTGGACATGGATGACTT<br>AGCTCCTCTCAATAAGGGCC	6
Col1a1	Collagen, type I, α 1	GACATGTTCAGCTTTGTGGACCTC<br>GGGACCCTTAGGCCATTGTGTA	7
Col3a1	Collagen, type III, α 1	GGTGGTTTTCAGTTCAGCTATGG<br>CTGGAAAGAAGTCTGAGGAATGC	3
Col6a3	Collagen, type VI α 3	TGATGGCACCTCTCAGGACTCT<br>TTGTCGGAGCCATCCAAAAG	8
Cybb	Cytochrome B-245 β	TGTGTCGAAATCTGCTCTCCTTT<br>AAAGTGAGGTTCCTGTCCAGTTGT	8
Fabp4	Fatty Acid Binding Protein 4	AAGGTGAAGAGCATCATAACCCT<br>TCACGCCTTTCATAACACATTCC	9
Fn1	Fibronectin	TCGCACTGGTAGAAGTTCCA<br>ATCATTTCATGCCAACCAGTT	10

Gapdh	Glyceraldehyde-3-phoshate	AACGACCCCTTCATTGACCTC<br>CTTCCCATTCTCAGCCTTGACT	3
Gpnmb	Glycoprotein nonmetastatic melanoma protein b	GAAGCCAGCATCTCAGGTTC<br>CTGAACACCGACCCAGTTTT	3
Il1β	Interleukin-1*β*	GGCTCATCTGGGATCCTCTC<br>TCATCTTTTGGGGTCCGTCA	6
Il6	Interleukin-6	CTGCAAGAGACTTCCATCCAGTT<br>GAAGTAGGGAAGGCCGTGG	2
Il10	Interleukin-10	CAGGGATCTTAGCTAACGGAAA<br>GCTCAGTGAATAAATAGAATGGGA<br>AC	11
Itgax	Integrin Subunit α X (also known as CD11c)	TTCTTCTGCTGTTGGGGTTTG<br>CAACCACCACCCAGGAACTAT	12
Lep	Leptin	GGTAGAGCCTTTGGGCTGTC<br>TAAGTGCTTCCATCGTGTGC	
Mrc1	Mannose Receptor C-Type 1 (also known as CD206)	CAAGGAAGGTTGGCATTTGT<br>CCTTTCAGTCCTTTGCAAGC	6
Nos2	Nitric Oxide Synthase 2	ACCCAAGGTCTACGTTCAGG<br>CGCACATCTCCGCAAATGTA	13
Plin2	Perilipin 2 (also known as Adipophilin)	GAAGAGAAGCATCGGCTACGA<br>GTCAGGTTGCGGGCGATA	
Pparg	Peroxisome Proliferator Activated Receptor γ	GAAAGACAACGGACAAATCACC<br>GGGGGTGATATGTTTGAACTTG	
Sdc4	Syndecan-4	CTCCTGGAAGGCAGATACTTCTC<br>GTGTCATCCAGATCTCCAGAACC	3
Slc2a4	Solute carrier family 2 member 4 (also known as Glut4)	ACATACCTGACAGGGCAAGG<br>CGCCCTTAGTTGGTCAGAAG	14
Tbp	TATA box-binding protein-like protein 1 (Tbpl1)	CCCTATCACTCCTGCCACACC<br>CGAAGTGCAATGGTGTTTAGGTC	3
Tgfβ1	Transforming growth factor β-1 proprotein	TGCGCTTGCAGAGATTAAAA<br>CTGCCGTACAACTCCAGTGA	15
Tnfα	Tumor necrosis factor	CCCTCACACTCAGATCATCTTCT<br>GCTACGACGTGGGCTACAG	16

SI Immunohistochemistry and Tissue Staining

Organs were fixed in 4% paraformaldehyde, washed twice in PBS and dehydrated in an increasing series of alcohol and embedded in fresh paraffin. Sections were cut with a rotary microtome, mounted on a SuperFrost slide and air-dried overnight at room temperature.

Paraffin sections were deparaffinized with xylene and rehydrated with a decreasing series of alcohol. Antigens were unmasked by boiling the sections in sodium citrate buffer (10 mM sodium citrate, 0.05% Tween-20, pH 6.0) for 20 min and washed with PBS. After incubation with 10% normal donkey serum in PBS for 10 min, the sections were incubated with the primary antibody in a humidified chamber overnight at 4 °C. If double staining was performed on the same section, each primary antibody was applied separately for one night. Plin2 primary antibody was labeled with Cy3-conjugated anti-guinea pig secondary antibody, Lgals3 primary antibody was labeled with Cy3-and AlexaFlour 488-conjugated anti-rat secondary antibody, Gpnmb primary antibody was labeled with Cy3-conjugated anti-goat secondary antibody, Mrc1 primary antibody was labeled with Cy3-conjugated anti-goat secondary antibody. The respective secondary antibodies (all dianova Jackson ImmunoResearch), diluted 1:300 in PBS, were applied also separately at room temperature for 2 h each in humidified chamber. Sections were mounted with nuclei staining 4, 6-diamidino-2-phenylindole (DAPI) in Vectashield mounting medium and covered with a cover slip.

Supplemental Figures

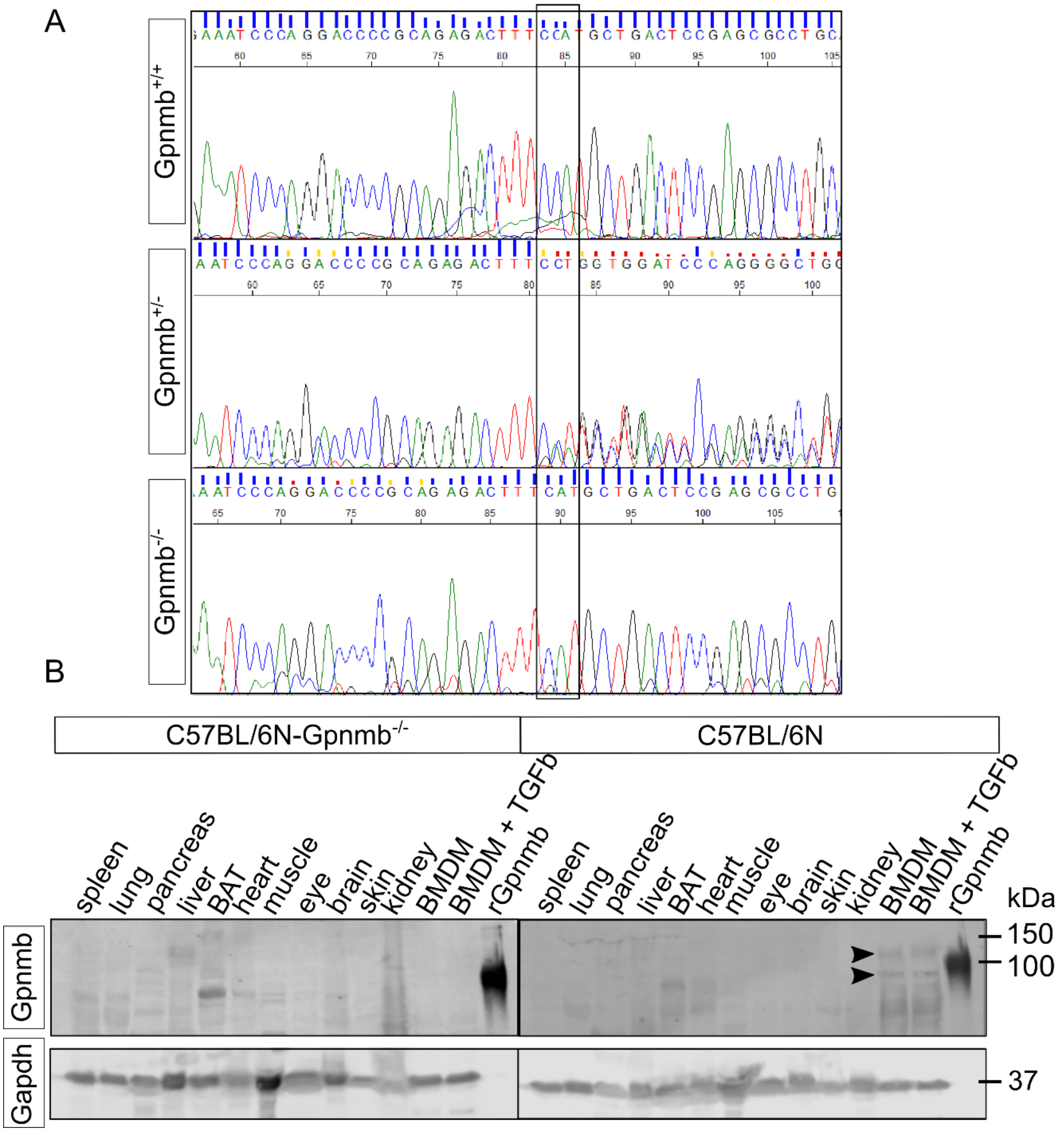

<u>Figure S1: Verification of the newly generated Gpnmb$^{-/-}$ strain.</u>

A) Sequencing of different C57BL/6N-mouse genotypes with 3' reverse primer. The complement of TTTCCAT is ATGGAAA, the second guanine is deleted. TTTCCAT is the wildtype, TTTCAT is the Gpnmb-knockout sequence. **B**) Verification by Western blot. Black arrow heads indicate specific Gpnmb signals. BMDMs were treated either with 10 ng/mL TGFβ or vehicle for 6 h. Cropped images, the respective full-length blots are presented in Figure S10. BAT: brown adipose tissue; BMDM: bone marrow-derived macrophages; rGpnmb: recombinant Gpnmb protein.

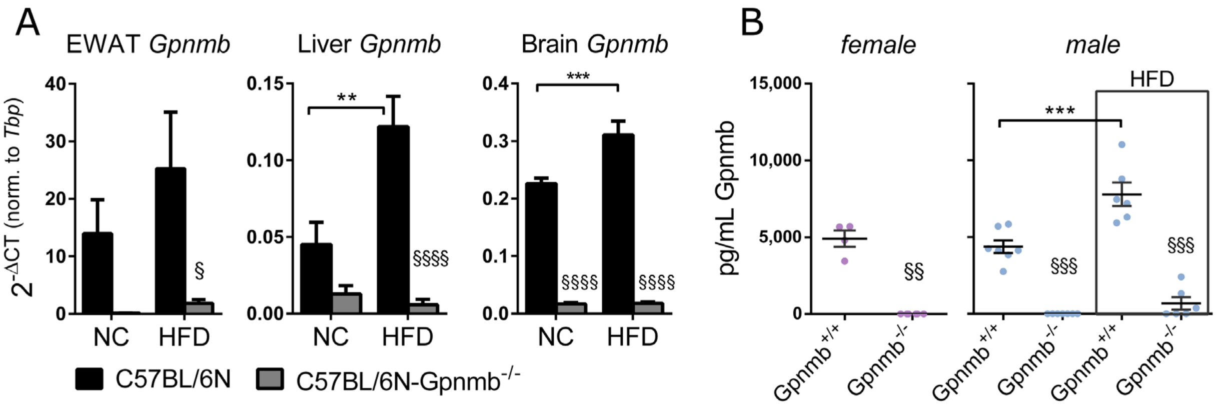

<u>Figure S2: Gpnmb expression of lean or HFD-fed, Gpnmb$^{-/-}$ and wildtype mice.</u>

A) Gpnmb mRNA levels of male mice, measured by qRT-PCR. **B**) Measurement of soluble Gpnmb in plasma by ELISA. NC: normal chow, HFD: high fat diet. n=6-7, mean ± SEM. Statistical differences were determined by a non-parametric t-test/Mann Whitney test for female animals of B, all remaining graphs by a Two-way ANOVA with Bonferroni post-hoc tests; ** p<0.01; *** p<0.001; **** p<0.0001; significant differences between genotypes: § p<0.05, §§ p<0.01; §§§ p<0.001; §§§§ p<0.0001.

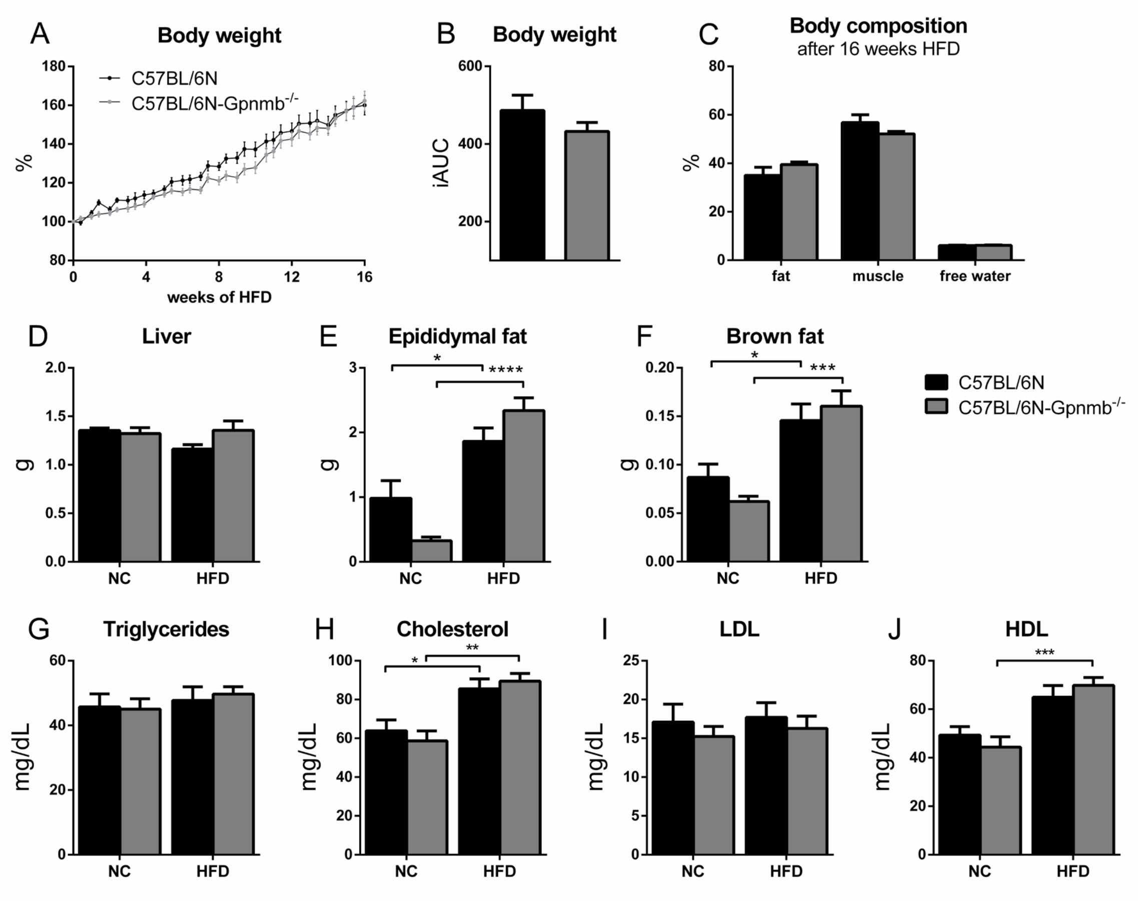

Figure S3: Impact of Gpnmb and 16 weeks of HFD on body weight, organ weight and plasma lipid parameters of lean or HFD-fed, male Gpnmb$^{-/-}$ and wildtype mice.

A) Body weight curve and **B**) integrated area under the curve of the body weight (iAUC). **C**) Body composition analysis at the end of the 16 weeks of HFD. **D-F**) Organ weight. **G-J**) Blood lipid parameters in plasma. NC: normal chow, HFD: high fat diet. n=6-7, mean ± SEM. Statistical differences were tested by a Two-way ANOVA with Bonferroni post-hoc tests (A, C-J) or by a non-parametric t-test/Mann Whitney test (B); * $p<0.05$; ** $p<0.01$; *** $p<0.001$; **** $p<0.0001$.

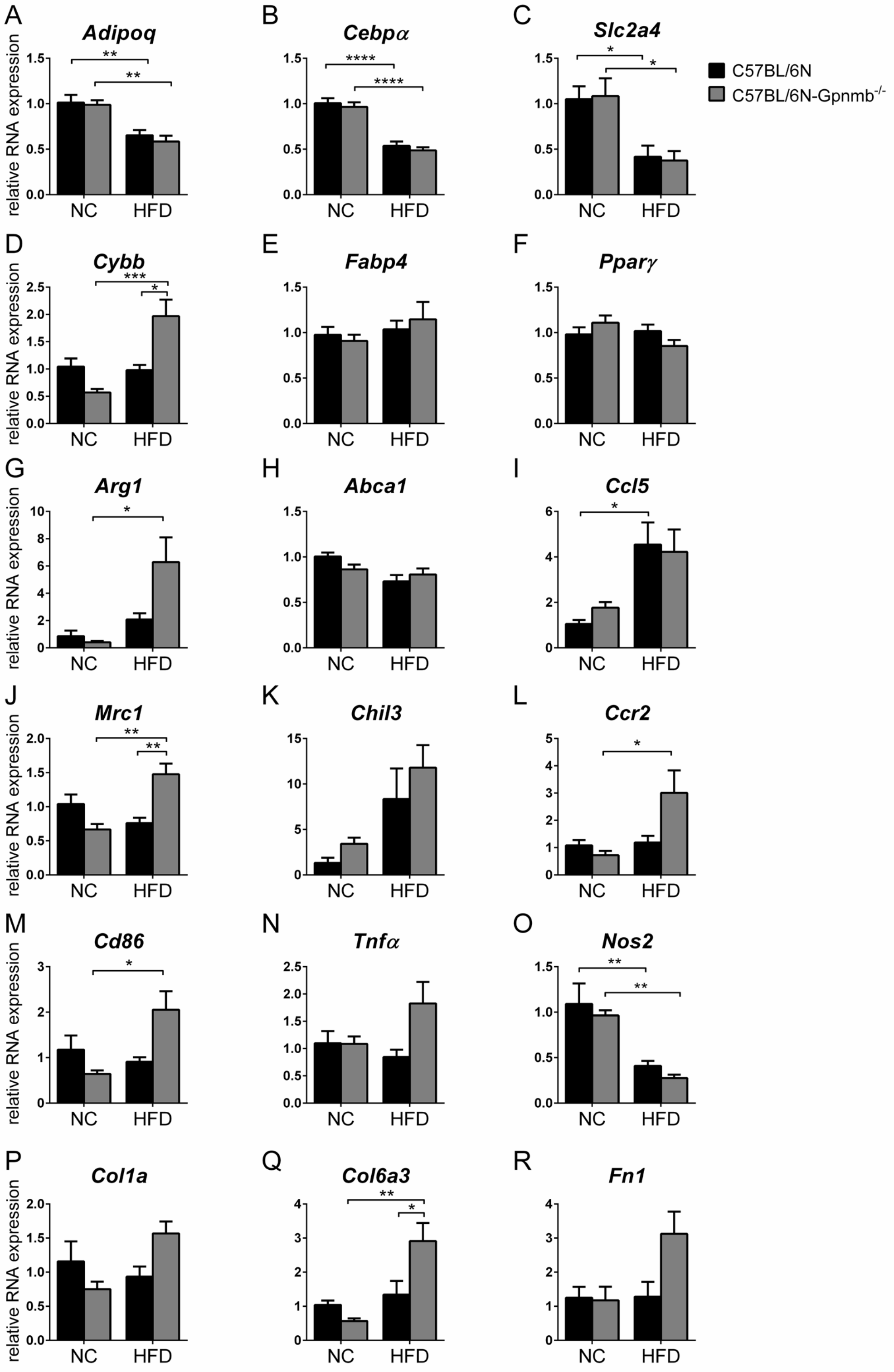

Figure S4: Inflammation status in epididymal adipose tissue of male lean or HFD-fed Gpnmb$^{-/-}$ and wildtype mice.

Transcript levels were measured by qRT-PCR. Relative RNA expression was calculated using the $2^{-\Delta\Delta Ct}$ method, normalized to the housekeeping genes and to NC-fed, wildtype animals. NC: normal chow, HFD: high fat diet. n=5-7, mean ± SEM. Statistical differences were determined by a Two-way ANOVA with Bonferroni post-hoc tests; * p<0.05; ** p<0.01, *** p<0.001, **** p<0.0001.

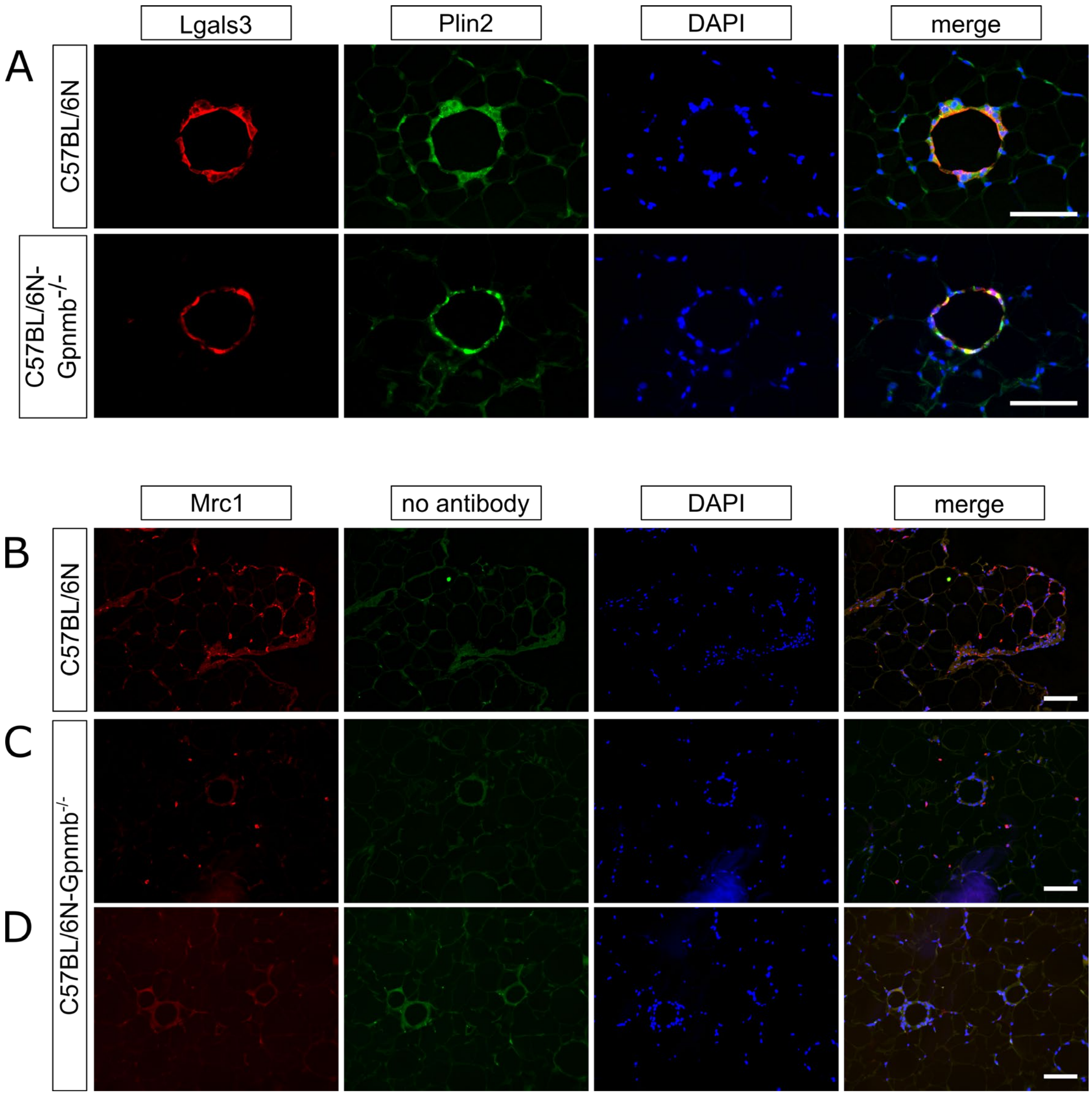

Figure S5: Immunhistological images of epididymal adipose tissue of HFD-fed, male Gpnmb$^{-/-}$ and wildtype mice.

A) Representative pictures of foamy macrophages in crown-like structures. **B-D**) Mrc1 localization in epididymal adipose tissue. The Mrc1-positive cells appeared isolated and only rarely localized to CLSs in both wildtype or Gpnmb$^{-/-}$ HFD-fed animals (**C, D**). The cells were unevenly distributed in the adipose tissue. **D**) shows an area that seems macrophage-rich but lacks Mrc1-positive cells. The green channel in **B-D** was used to visualize the autofluorescence of erythrocytes. Immunohistological stainings of 7 µm adipose tissue sections. Scale bar: 100 µm.

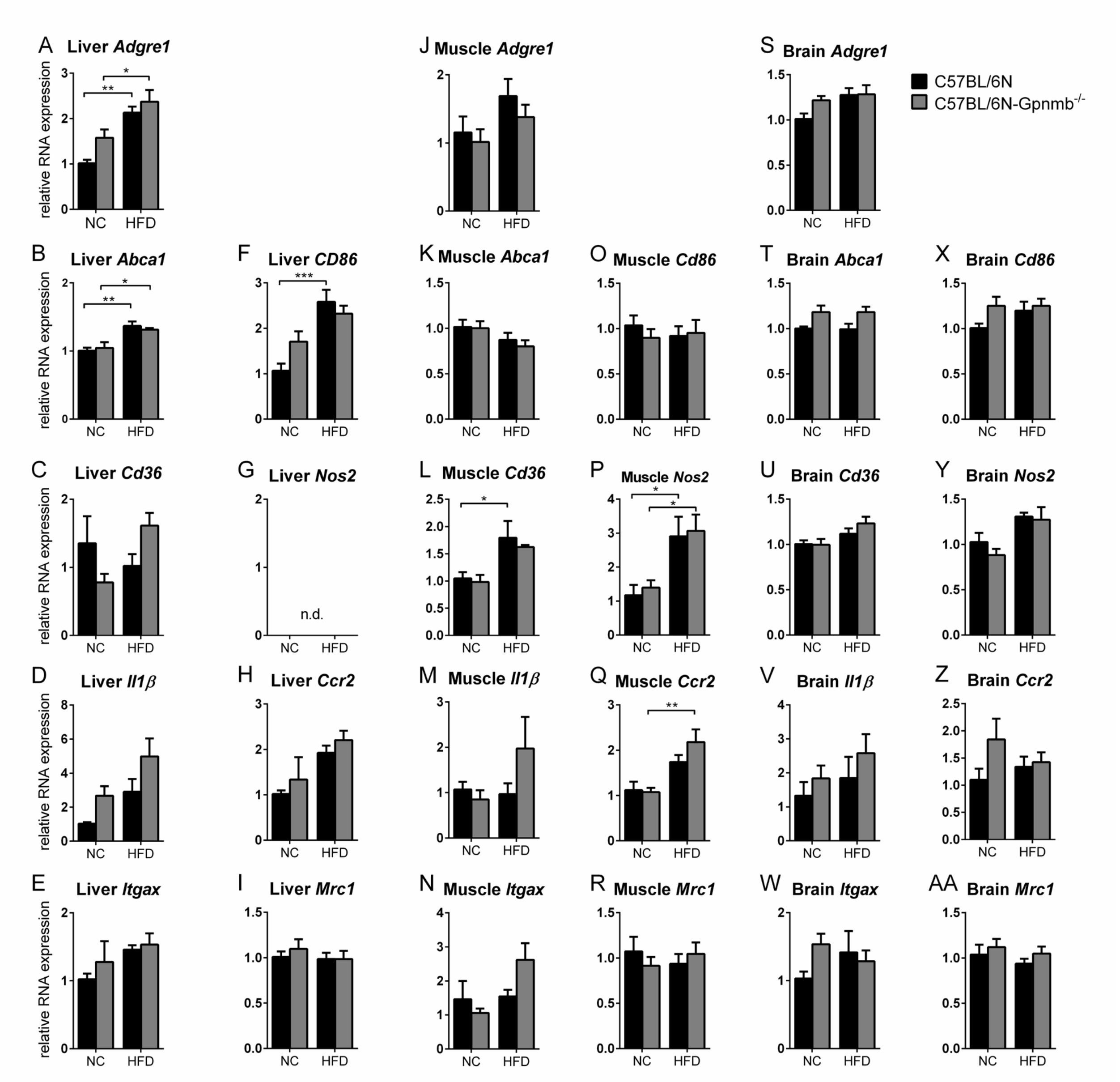

Figure S6: Inflammation status in liver, muscle and brain tissues of male lean or HFD-fed Gpnmb$^{-/-}$ and wildtype mice.

Transcript levels were measured by qRT-PCR. Relative RNA expression was calculated using the $2^{-\Delta\Delta Ct}$ method, normalized to the housekeeping genes and to NC-fed, wildtype animals. n.d.: not detected, NC: normal chow, HFD: high fat diet. n=6-7, mean ± SEM. Statistical differences were determined by a Two-way ANOVA with Bonferroni post-hoc tests; * p<0.05; ** p<0.01, *** p<0.001.

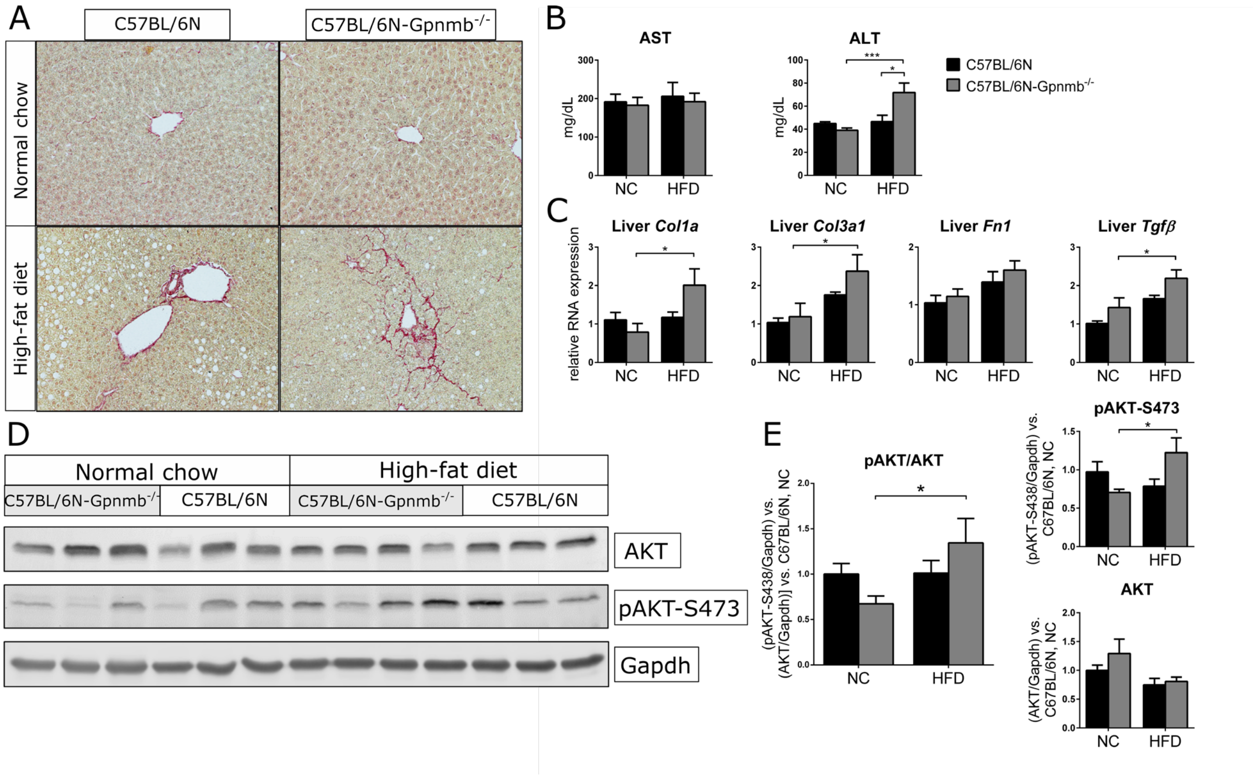

Figure S7: Analysis of hepatic fibrosis in male lean or HFD-fed Gpnmb-/- and wildtype mice.

A) Representative pictures of Sirius red staining of 5 µm liver tissue sections. **B**) Liver damage markers in plasma tissue. **C**) Fibrotic mRNA levels in liver, measured by qRT-PCR. Relative RNA expression was calculated using the $2^{-\Delta\Delta Ct}$ method, normalized to a housekeeping gene and to NC-fed, wildtype animals. **D**) Representative Western blot and **E**) quantification of Western blots of pan-AKT and AKT phosphorylated at serine 473 (pAKT). Cropped images, the respective full-length blots are presented in Figure S11. ALT, alanine aminotransferase, AST: aspartate aminotransferase, NC: normal chow, HFD: high fat diet. n=6-7, mean ± SEM. Statistical differences were determined by a Two-way ANOVA with Bonferroni post-hoc tests; * p<0.05; *** p<0.001.

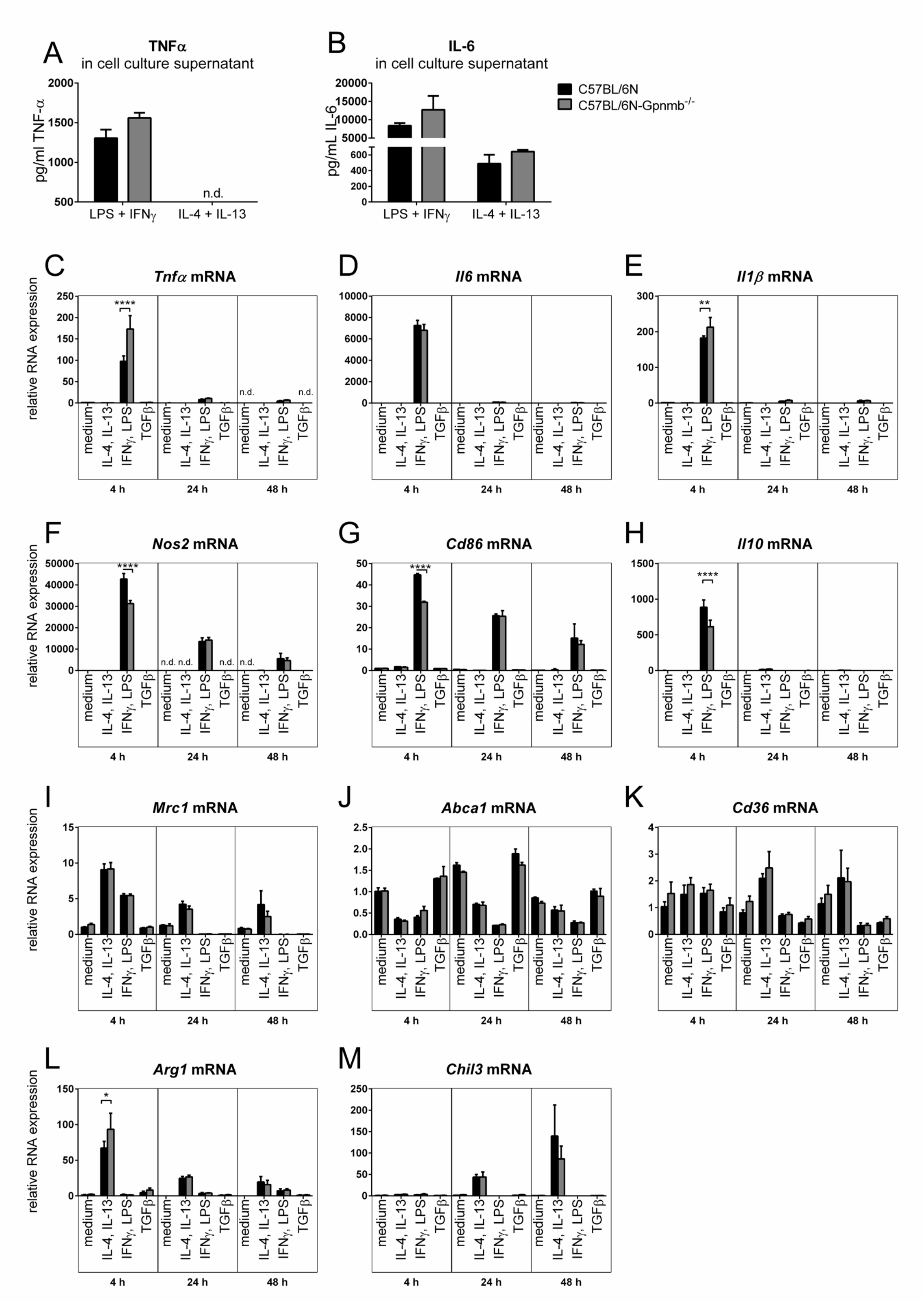

Figure S8: Inflammation status of Gpnmb-/- and wildtype macrophages.

Cells were stimulated with IL-4 and IL-13, or LPS and IFNγ. **A, B**) Inflammatory cytokine release into the supernatant of macrophages treated for 48 h, measured by ELISA. No statistical difference was detected between genotypes using a non-parametric t-test/Mann Whitney test. **C-M**) Transcript levels of differently

polarized macrophages were measured by qRT-PCR. Relative RNA expression was calculated using the $2^{-\Delta\Delta Ct}$ method, normalized to *Tbp* and to the condition of unstimulated macrophages derived from wildtype animals. Statistical differences were determined by a Two-way ANOVA with Bonferroni post-hoc tests. n.d.: not detected. n=3, meaning BMDMs from three individual mice per group, mean ± SEM. * $p<0.05$; ** $p<0.01$, **** $p<0.0001$.

Full-length Western Blots

Figure S9: Full-length blots of Figure 3D (Gpnmb mRNA and protein levels of differently polarized Gpnmb-/-- and wildtype macrophages.)

Anti-Gpnmb in Figure 3D:

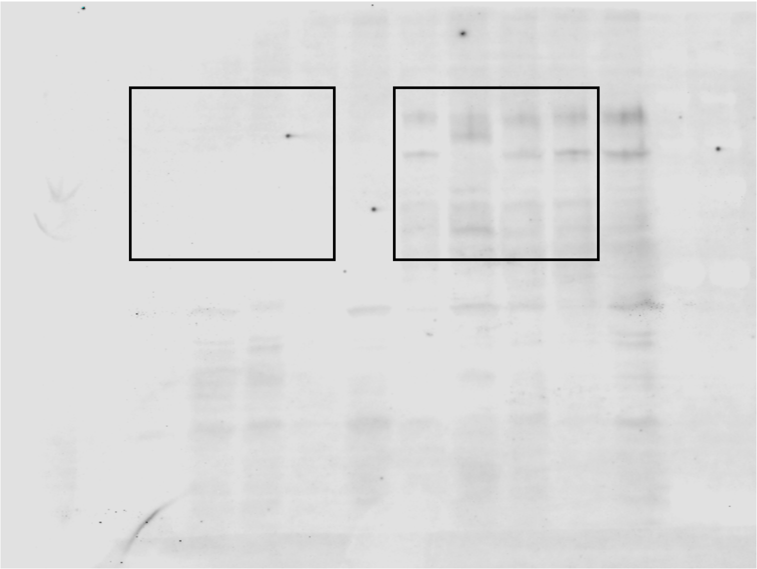

→ *Quantification of Gpnmb antibody signal:*

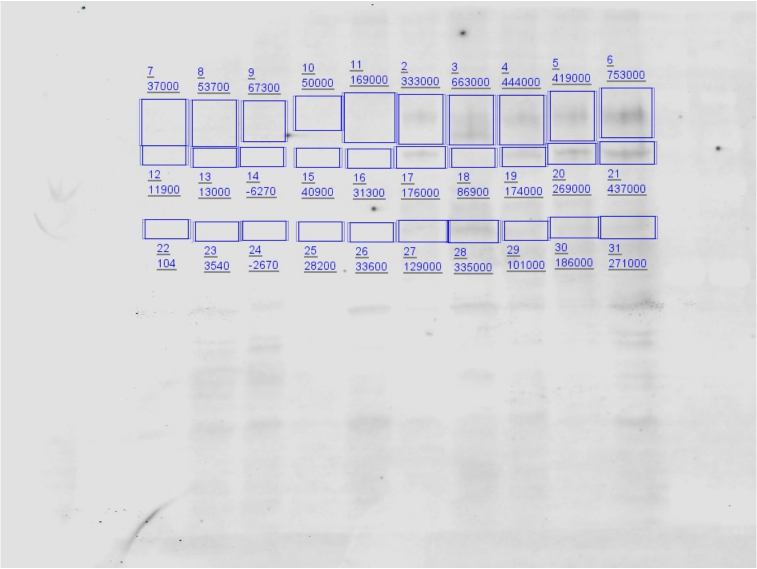

Anti-Gapdh antibody signal in Figure 3D

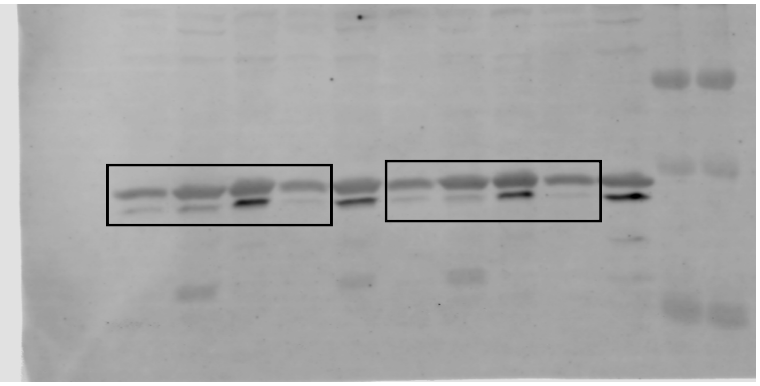

→ *Quantification of Gapdh antibody signal:*

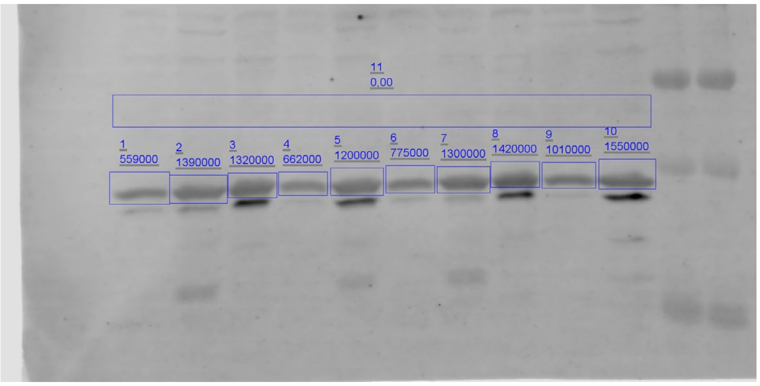

Figure S10: Full-length blots of Figure S1B (Verification of the newly generated Gpnmb$^{-/-}$ strain.)

Anti-Gpnmb antibody signal in Figure S1B

C57BL/6N-Gpnmb$^{-/-}$

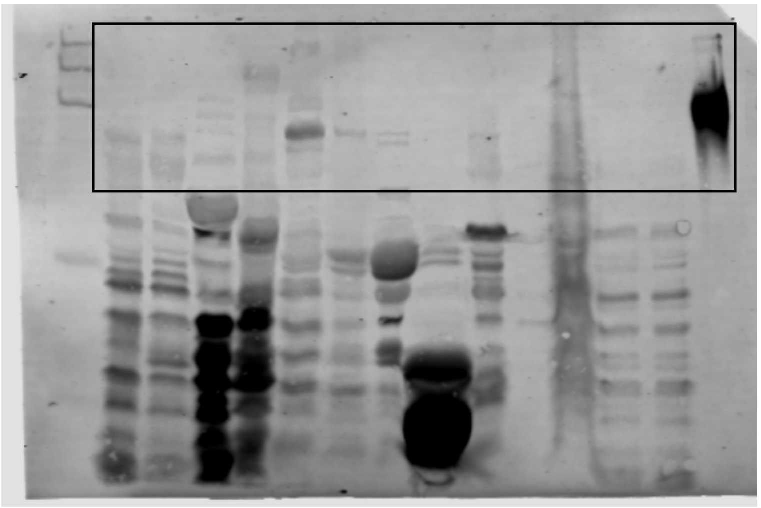

C57BL/6N

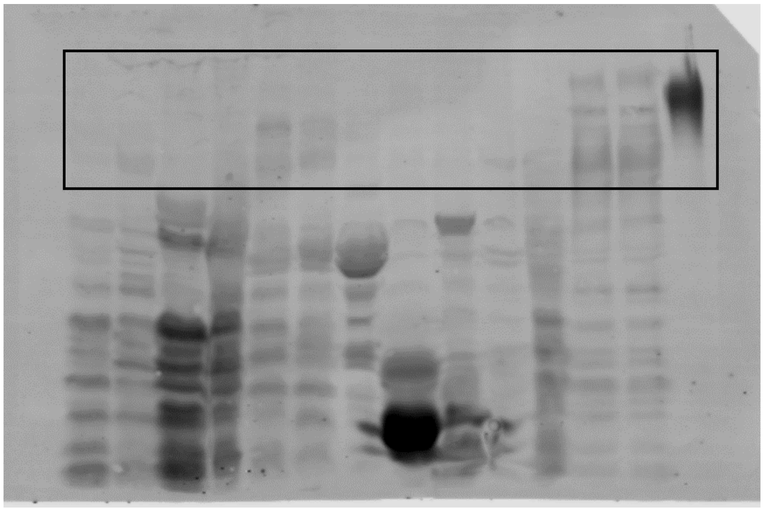

Anti-Gapdh antibody signal in Figure S1B

C57BL/6N-Gpnmb$^{-/-}$

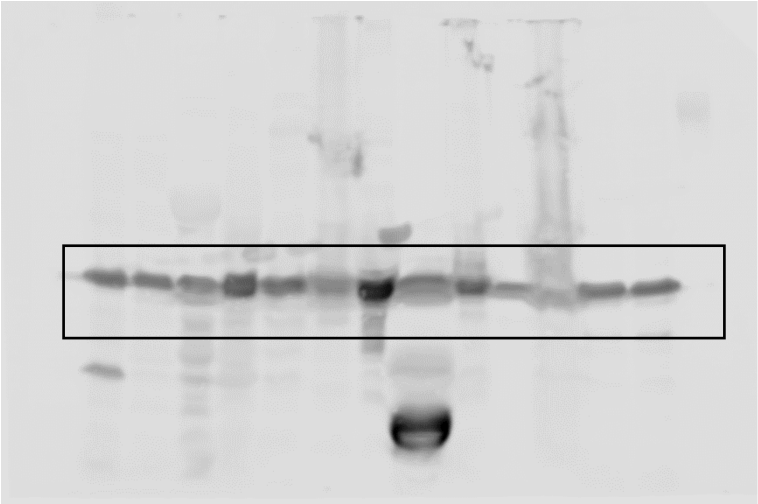

C57BL/6N

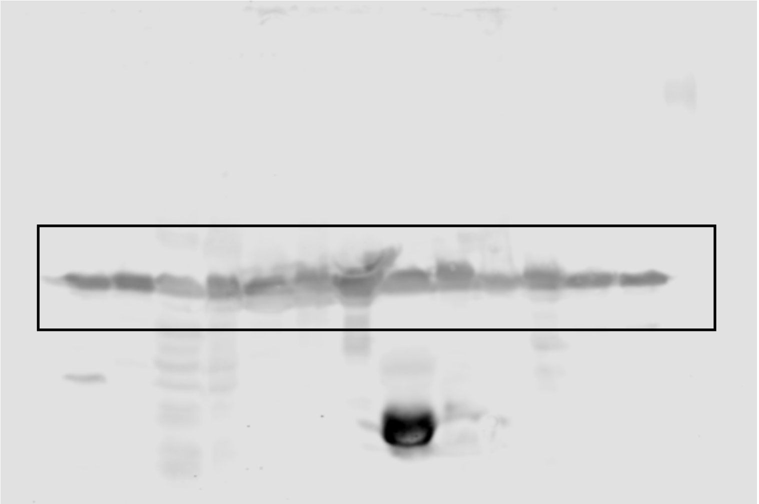

Figure S11: Full-length blots of Figure S7D (Analysis of hepatic fibrosis in male lean or HFD-fed Gpnmb$^{-/-}$ and wildtype mice.)

Anti-AKT pan antibody signal in Figure S7D

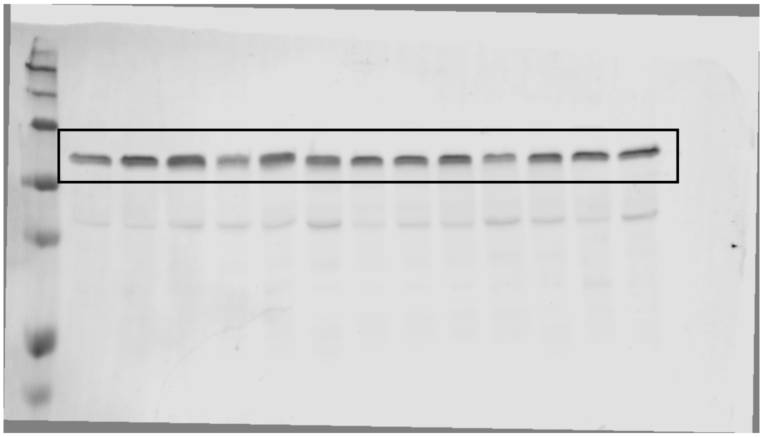

→ *Quantification of AKT antibody signal:*

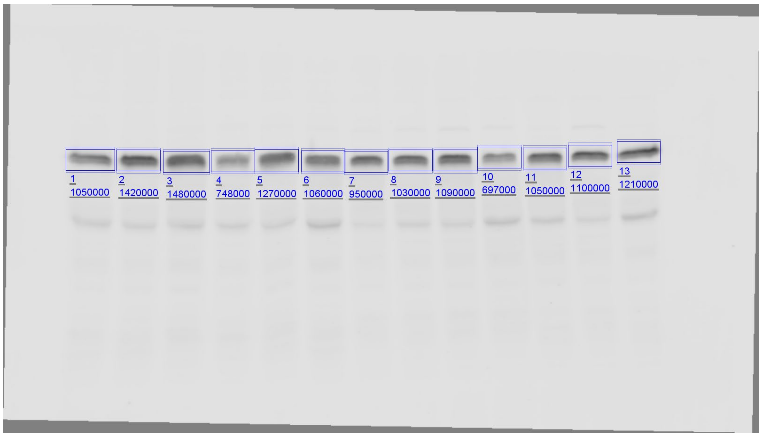

Anti-pAKT-S473 antibody signal in Figure S7D

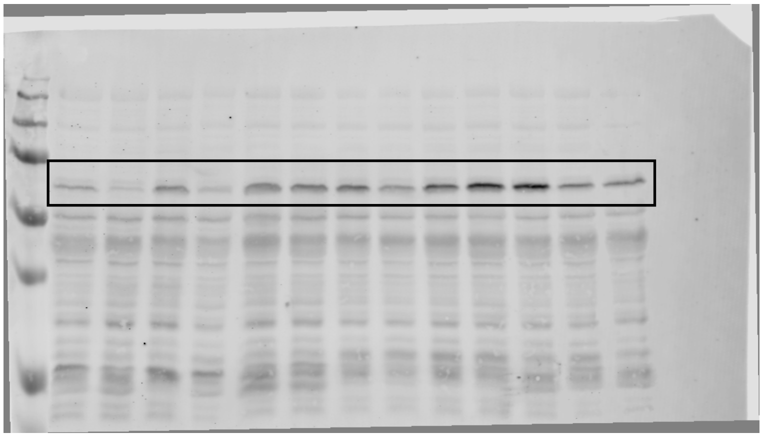

→ Quantification of pAKT-S473 antibody signal:

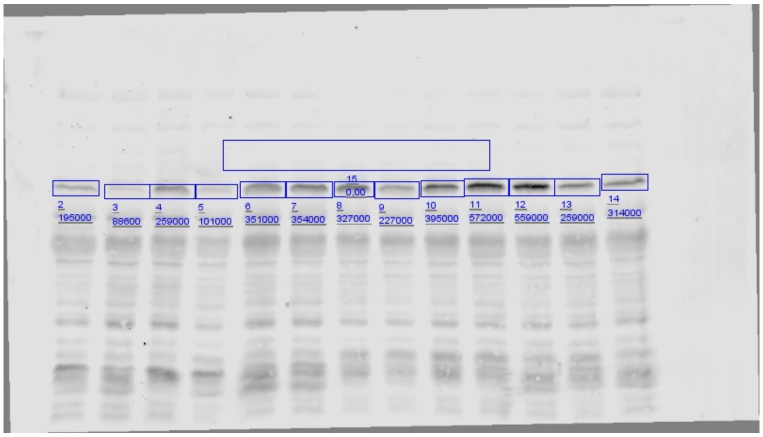

Anti-Gapdh antibody signal in Figure S7D

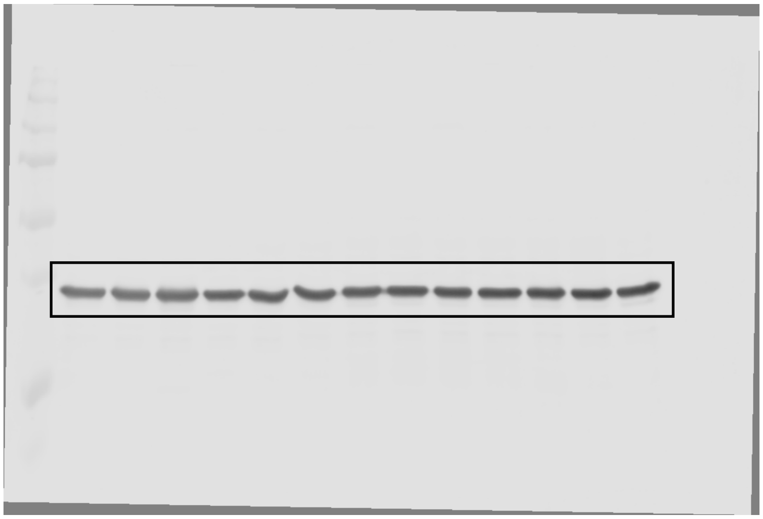

→ Quantification of Gapdh antibody signal:

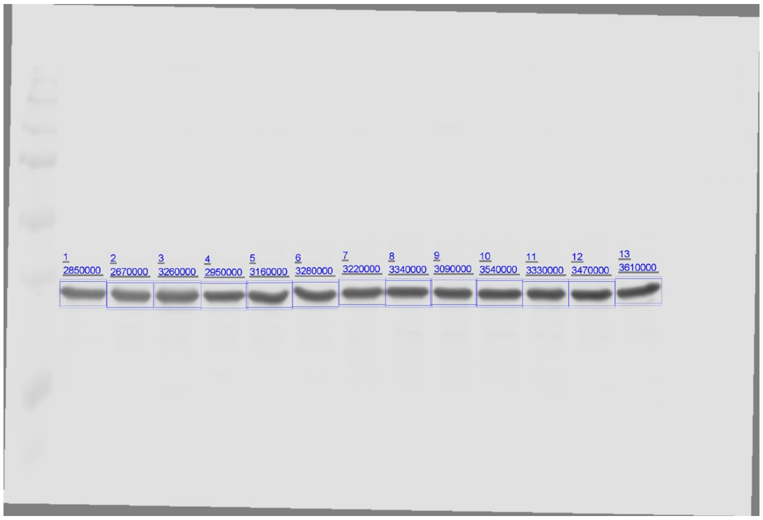

References

1. Cong, L. *et al.* Multiplex genome engineering using CRISPR/Cas systems. *Science* **339**, 819–823 (2013).
2. Rousselle, A. *et al.* CXCL5 limits macrophage foam cell formation in atherosclerosis. *J Clin Invest* **123**, 1343–1347 (2013).
3. Järve, A. *et al.* Adverse left ventricular remodeling by glycoprotein nonmetastatic melanoma protein B in myocardial infarction. *FASEB J.* **31**, 556–568 (2017).
4. Yu, H. *et al.* Hypothalamic POMC deficiency increases circulating adiponectin despite obesity. *Mol Metab* **35**, 100957 (2020).
5. Zhang, X., Liu, F., Bai, P., Dong, N. & Chu, C. Identification of key genes and pathways contributing to artery tertiary lymphoid organ development in advanced mouse atherosclerosis. *Mol Med Rep* **19**, 3071–3086 (2019).
6. Cavalcante, P. A. M. *et al.* Nephropathy in Hypertensive Animals Is Linked to M2 Macrophages and Increased Expression of the YM1/Chi3l3 Protein. *Mediators Inflamm* **2019**, 9086758 (2019).
7. Feng, X. *et al.* Injectable cartilaginous template transformed BMSCs into vascularized bone. *Scientific Reports* **8**, 8244 (2018).
8. Lancha, A. *et al.* Osteopontin Deletion Prevents the Development of Obesity and Hepatic Steatosis via Impaired Adipose Tissue Matrix Remodeling and Reduced Inflammation and Fibrosis in Adipose Tissue and Liver in Mice. *PLoS One* **9**, (2014).
9. Zhao, Y. *et al.* Divergent functions of endotrophin on different cell populations in adipose tissue. *Am J Physiol Endocrinol Metab* **311**, E952–E963 (2016).
10. Wang, S. *et al.* MR Elastography-Based Assessment of Matrix Remodeling at Lesion Sites Associated With Clinical Severity in a Model of Multiple Sclerosis. *Front Neurol* **10**, (2020).
11. Wang, H. *et al.* HISTONE DEACETYLASE INHIBITOR LAQ824 AUGMENTS INFLAMMATORY RESPONSES IN MACROPHAGES THROUGH TRANSCRIPTIONAL REGULATION OF IL-10. *J Immunol* **186**, 3986–3996 (2011).
12. Uchida, K. *et al.* CD11c+ macrophages and levels of TNF-α and MMP-3 are increased in synovial and adipose tissues of osteoarthritic mice with hyperlipidaemia. *Clin Exp Immunol* **180**, 551–559 (2015).
13. Cho, C.-W. *et al.* Cynanchum wilfordii Polysaccharides Suppress Dextran Sulfate Sodium-Induced Acute Colitis in Mice and the Production of Inflammatory Mediators from Macrophages. *Mediators Inflamm* **2017**, 3859856 (2017).
14. Montel-Hagen, A. *et al.* The Glut1 and Glut4 glucose transporters are differentially expressed during perinatal and postnatal erythropoiesis. *Blood* **112**, 4729–4738 (2008).
15. Yang, L. *et al.* Sphingosine 1-Phosphate Receptor 2 and 3 Mediate Bone Marrow-Derived Monocyte/Macrophage Motility in Cholestatic Liver Injury in Mice. *Sci Rep* **5**, (2015).
16. Sanson, M., Distel, E. & Fisher, E. A. HDL Induces the Expression of the M2 Macrophage Markers Arginase 1 and Fizz-1 in a STAT6-Dependent Process. *PLoS ONE* **8**, e74676 (2013).
